# Hierarchical nickel valence gradient stabilizes high-nickel content layered cathode materials

**DOI:** 10.1038/s41467-021-22635-w

**Published:** 2021-04-20

**Authors:** Ruoqian Lin, Seong-Min Bak, Youngho Shin, Rui Zhang, Chunyang Wang, Kim Kisslinger, Mingyuan Ge, Xiaojing Huang, Zulipiya Shadike, Ajith Pattammattel, Hanfei Yan, Yong Chu, Jinpeng Wu, Wanli Yang, M. Stanley Whittingham, Huolin L. Xin, Xiao-Qing Yang

**Affiliations:** 1 Chemistry Division, Brookhaven National Laboratory, Upton, NY USA; 2 Applied Materials Division, Argonne National Laboratory, Lemont, IL USA; 3 Department of Physics and Astronomy, University of California, Irvine, CA USA; 4 Center for Functional Nanomaterials, Brookhaven National Laboratory, Upton, NY USA; 5 National Synchrotron Light Source II, Brookhaven National Laboratory, Upton, NY USA; 6 Advanced Light Source, Lawrence Berkeley National Laboratory, Berkeley, CA USA; 7 Materials Science and Engineering, Binghamton University, Binghamton, NY USA; 8 Present Address: National Synchrotron Light Source II, Brookhaven National Laboratory, Upton, NY USA

**Keywords:** Batteries, Batteries, Batteries

## Abstract

High-nickel content cathode materials offer high energy density. However, the structural and surface instability may cause poor capacity retention and thermal stability of them. To circumvent this problem, nickel concentration-gradient materials have been developed to enhance high-nickel content cathode materials’ thermal and cycling stability. Even though promising, the fundamental mechanism of the nickel concentration gradient’s stabilization effect remains elusive because it is inseparable from nickel’s valence gradient effect. To isolate nickel’s valence gradient effect and understand its fundamental stabilization mechanism, we design and synthesize a LiNi_0.8_Mn_0.1_Co_0.1_O_2_ material that is compositionally uniform and has a hierarchical valence gradient. The nickel valence gradient material shows superior cycling and thermal stability than the conventional one. The result suggests creating an oxidation state gradient that hides the more capacitive but less stable Ni^3+^ away from the secondary particle surfaces is a viable principle towards the optimization of high-nickel content cathode materials.

## Abstract (web summary)

High-nickel content cathode materials suffer issues of structural and surface instability. Herewith authors show that introduction of a nickel valence gradient enhances the thermal and cycle stability of high-nickel content cathode materials.

## Introduction

To meet the demand of high energy density, low cost, and long cycle life lithium-ion batteries for electric vehicles, high-nickel-content layered cathode materials have attracted intensive research interests for their higher capacity compared with widely commercialized materials such as LiCoO_2_ (LCO) and LiNi_1/3_Mn_1/3_Co_1/3_O_2_ (NMC-333). By increasing nickel content in NMC-333, high-nickel cathode materials increase capacity and reduce cost comparing with LCO and NMC-333. Higher nickel content is able to provide higher specific capacity because Ni^2+^ can be fully oxidized to Ni^4+^. In contrast, it is hard to oxidize Co to higher valence state than Co^3.6+^ without oxygen redox activity^1,2^. However, there are remaining problems for this type of materials, such as structure deterioration^3–5^, unclear surface chemistry, thermal instability^6–8^, and capacity fading^9,10^. For example, Ni^3+^ is chemically unstable and tends to be reduced to Ni^2+^, which has an ionic radius close to that of Li^+^ ^11^, Therefore Ni^2+^ has the tendency to migrate into the lithium layer when lithium is extracted from the lattice. This migration can induce irreversible structure changes and capacity fading^12^. It also has been reported that the anisotropic volume expansion of the layered materials during delithiation can cause the formation of micro-cracks^13,14^ (crack size >100 nm) and nanocracks^15,16^ (crack size <100 nm) at the secondary and primary particle levels. These cracks block electrical contact which in turn reduce the utilization of active materials.

The surface/near-surface chemistry of layered oxide materials is another determining factor for the cathode’s performance. It is well known that lithium nickel-manganese-cobalt oxide (NMC) cathode materials undergo surface degradation to form rock-salt structure or disordered spinel phase on the primary particle surfaces, which subsequently causes the impedance buildup and induces further irreversible structure degradation originated from the surfaces^17^. Therefore, how to modify the surface in order to reduce the undesirable interaction of high-nickel cathode materials with the electrolyte, especially during high voltage charging is still a grand challenge. Surface coating, concentration gradient, and core–shell structure are the most widely adopted strategies to improve the material surface stability^18–21^. It has been reported by Amine, Sun, and their colleagues that introducing Ni/Mn-concentration gradient is particularly effective for improving the cycle life of NMC materials^20,22–24^; however, the factors responsible for the improved stability have not been fully understood. It has been hypothesized that the reduced nickel atomic concentration on the surface can reduce the undesired surface reactivity but recent reports suggest that it is the high valence states (Ni^3+^ and Ni^4+^), not the concentration of Ni, that causes the instability. This suggests that reducing the amount of chemically unstable Ni^3+^ and Ni^4+^ at and near the surface is the key to obtain the surface stability. It has been shown is possible to introduce Ni^2+^ ions as ‘pillars’ in the lithium layer^25^, but they generally reduce capacity and impede lithium diffusion. So far, it still remains challenging to obtain a perfect layered structure and avoid sacrificing electrochemical capacity while decreasing the amount of unstable Ni^3+^ in high-Ni-content cathode material synthesis.

In this work, to pinpoint the surface stabilization mechanism(s) for high-nickel-content materials and to find approaches to optimize the material, we synthesize and investigate a model system that has a valence-gradient hierarchical secondary particle architecture. Different from concentration-gradient or core–shell-structured NMC materials where Mn/Ni concentration varies throughout the particle, our newly designed high-Ni-content cathode material has a uniform Ni:Mn:Co = 8:1:1 chemical composition throughout the secondary particle. However, in this architecture, there is a nickel-valence-state gradient along the radial direction of the secondary particle. In this architecture, for the discharged state, a large amount of Ni ions at the surface and in the near-surface region (~ top 900-nm surface region) are at the Ni^2+^ state correlated with oxygen deficiency, while in the center bulk are at closer to the Ni^3+^ state. Using this model structure for our mechanistic study, we are able to separate the effects of the nickel-valence state from nickel concentration. Electrochemical testing shows that, with the help of Ni-valence gradience only, our valence-gradient LiNi_0.8_Mn_0.1_Co_0.1_O_2_ (VG-NMC811) shows improved capacity retention than the conventional NMC811 material. Our transmission electron microscopy (TEM) and synchrotron-based nanoprobe X-ray imaging studies show that, after extended cycling, the nickel oxidation state gradient and the surface structures are well maintained. Also, very interestingly, our results show that after cycling, nanopores are formed with a higher population in the bulk center rather than on the surfaces and the near-surface region of the material, indicating that the surface-initiated degradation in this material system is successfully suppressed. All of these suggest that nickel-valence gradient plays an important role in surface stabilization in high-nickel-content material.

## Results and discussion

### Characterization of pristine VG-NMC811

**Fig. 1:**
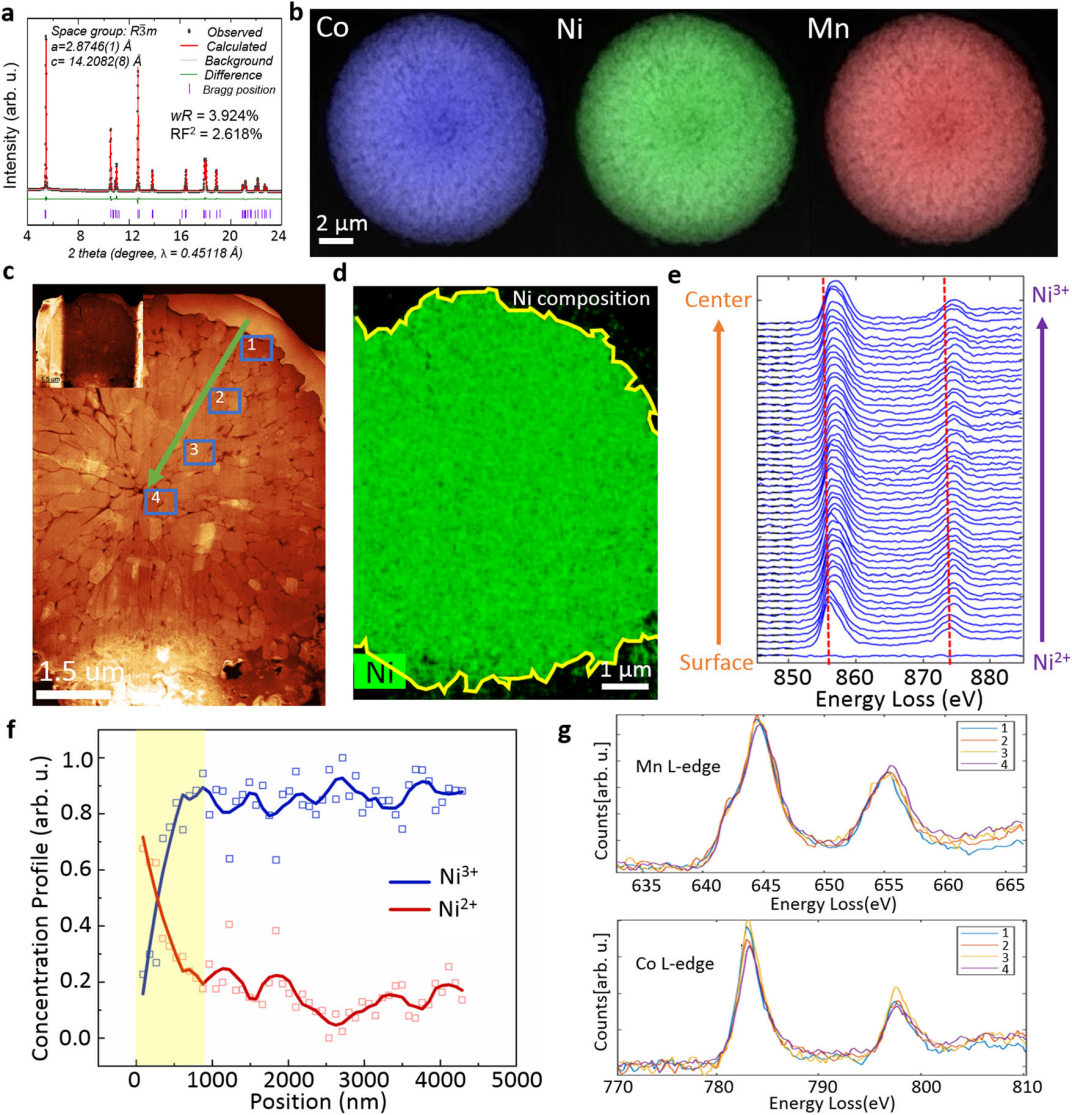
Structural and chemical characterization of the pristine valence gradient NMC811 material (VG-NMC811). **a** Rietveld refinement of pristine VG-NMC811. **b** X-ray fluorescence quantitative mapping of Ni, Mn, and Co showing uniform transition metal distribution across the whole secondary particle. **c** High-angle annular dark-field (HAADF) scanning transmission electron microscopy (STEM) images of a cross-sectional slice of VG-NMC811 secondary particle prepared by focused ion beam (FIB). **d** STEM-energy-dispersive X-ray spectroscopy (EDs) quantitative mapping of Ni. **e** The electron energy loss spectra (EELS) of Ni L_2,3_-edge across the surface and the center. **f** The Ni^2+^ and Ni^3+^ concentrations along the line profile extracted from spectra in (**e**). **g** The EELS spectra of Mn L-edge and Co L-edge integrated in the four squared regions in (**c**).

In this study, the pristine VG-NMC811 material is prepared by calcination of LiOH and a concentration-gradient type hydroxide precursor, which consists of Ni_0.9_Co_0.05_Mn_0.05_(OH)_2_ in the particle core and Ni_0.33_Mn_0.33_Co_0.33_(OH)_2_ on the particle surface. The baseline conventional NMC811 material is prepared using the same method except that the hydroxide precursor is compositionally homogeneous. Details of the synthesis process are in the “Methods” section and the further formation mechanism of VG-NMC811 will be discussed later in this paper. The powder X-ray diffraction (XRD) data in Fig. 1a shows that the pristine VG-NMC material has a typical O3-type layered structure (*R*$\bar{3}$*m* space group) without any impurity phases (also see Supplementary Fig. 1 for the atomic-resolution image of the O3-type lattice). Based on the Rietveld refinement (Supplementary Table 1), the percentage of Ni^2+^ in the Li layer is about 3.0% in the pristine VG-NMC811 material. The elemental distribution and composition are characterized by high-spatial resolution hard X-ray nanoprobe (HXN) imaging in conjunction with X-ray fluorescence (XRF) imaging. Figure 1b and Supplementary Fig. 2 show the high-resolution XRF mapping of Mn, Co, and Ni of a whole secondary particle. They show that all three transition metals are uniformly distributed. Figure 1c shows a high-angle annular dark-field scanning transmission electron microscopy (HAADF-STEM) image of a thin cross-section of a pristine VG-NMC811 secondary particle prepared by a dual-beam focused ion beam (FIB) instrument. It shows the secondary particle is composed of aggregated sub-micron-sized primary particles and the primary particles are closely packed with inter-granular boundaries between each other. To further confirm that the composition is uniform at the nanoscale, we also used STEM-energy-dispersive X-ray spectroscopy (EDS) mapping to map the elemental distribution of the transition metals. As shown in the STEM-EDS mapping of Ni, Mn, and Co composition in Fig. 1d and Supplementary Fig. 3 the Ni, Mn, and Co composition are nearly constant throughout the thin cross-sectional slice cut through a secondary particle, which is imaged in Fig. 1c. The quantification result of EDS mapping shows that the atomic contents of Ni, Mn, and Co are 78.14%, 10.63%, and 11.23%, respectively, which is in good agreement with the nominal 8:1:1 composition. STEM-EDS mapping and the composition analysis results of three other VG-NMC811 particles are provided in Supplementary Fig. 4 and Supplementary Table 2 and they show that the variation of Ni composition is on the order of 1–2% which is considered negligible because it is less than the error bar of our measurements. Combining with the XRF results in Supplementary Fig. 2, it is statistically reliable to conclude that the Ni composition is uniform throughout the secondary particle with a variation at or below the 2% uncertainty level.

We also performed spatially resolved electron energy loss spectroscopy (STEM-EELS) to probe the oxidation state of the transition metals. The EELS spectra collection along the marked green arrow in Fig. 1c is shown in Fig. 1e. The peak position of Ni L_3_ edge has a clear shift to higher energy as the probed spot moves from the surface to the center of a particle. It indicates a gradual increase of Ni-valence state. To determine the Ni-valence state, we perform a nonlinear least-square fit of each Ni L_2,3_ spectrum by a linear combination of the normalized Ni^2+^ and Ni^3+^ reference spectra^26^. The fitting coefficient represents the concentration of Ni^2+^ and Ni^3+^. As shown in Fig. 1f, the Ni^2+^ concentration is high at the surface and decreases rapidly within 900 nm in the near-surface region and gradually decreases toward the center while the Ni^3+^ concentration increases accordingly. It is worthwhile to notice that even though there is a large portion of Ni^2+^ existing in the 900-nm surface region, the material is well maintained in layered structure evidenced by the XRD results in Fig. 1a and Supplementary Table 1. At the same time, the EELS near-edge fine structures of Mn and Co (Fig. 1g) collected from the four marked areas in Fig. 1a show no chemical shift and little shape change, indicating both Mn and Co valence remain unchanged from the surface to the center of the particle. In addition, as shown in Supplementary Fig. 5, we also plotted the elemental concentration of oxygen showing that oxygen is deficient in the near-surface region where Ni^2+^ composition is high. During the acquisition of the EELS data, we have used the sub-pixel scanning technique to spread the dose over a 20–40 nm area for the acquisition of each spectrum to avoid radiation damage.

To verify the STEM-EELS results, we have also performed synchrotron radiation-based two-dimensional X-ray absorption near-edge structure (2D-XANES) mapping of the VG-NMC811 secondary particles using transmission X-ray microscopy (TXM). The Ni^2+^ composition maps of eight secondary particles are shown in Supplementary Fig. 6. It is evident that there is a higher concentration of Ni^2+^ in near-surface regions of the secondary particle which agrees very well with our STEM-EELS results. Because hard X-ray 2D-XANSE mapping has very little radiation damage during data collection and it was conducted under the ambient condition, it is very unlikely that the Ni^2+^ gradient is cause by radiation induced reduction. The TXM results provide additional support to the reliability of our STEM-EELS results. Therefore, we can conclude that Ni-valence gradient is truly present in the VG-NMC811 secondary particles.

However, what is the origin of the Ni^2+^ gradient? Because the Ni concentration remains at 80% throughout the particle, the Ni^2+^ valence gradient is not caused by the cation composition. As shown in Supplementary Fig. 5, the Ni^2+^ is highly correlated with oxygen deficiency, which suggest that there might be oxygen vacancies in the near-surface volume of the secondary particles, i.e., ${\rm{LiM}}{{\rm{O}}}_{2-{\rm{\delta }}}$. The Ni^2+^ gradient could also be a result of disordered rock-salt structure formation, i.e., ${\rm{L}}{{\rm{i}}}_{1-{\rm{x}}}{{\rm{M}}}_{1+{\rm{x}}}{{\rm{O}}}_{2}$. Unfortunately, Li composition is not quantifiable by EELS because its K edge overlaps with Mn and Co M edges, we are not able to prove or disprove the formation of disordered rock salt. In order to evaluate the relative amount of the rock-salt phase if any, we carried out the structural refinement of the pristine VG-NMC811 material with two phases (i.e., ${\rm{L}}{\rm{i}}_{1-{\rm{x}}}{\rm{M}}_{1+{\rm{x}}}{\rm{O}}_{2}={\rm{L}}{\rm{i}}_{1-{\rm{x}}}{\rm{M}}_{1-{\rm{x}}}{\rm{O}}_{2\left(1-{\rm{x}}\right)}({\rm{O}}3\ {\rm{phase}})\,+{\rm{M}}_{\rm{x}}{\rm{O}}_{2{\rm{x}}}({\rm{{rock}}\ {salt}\ {phase}})$). Comparing with the single phase model, the goodness of fitting only slightly improved but the phase fraction of rock salt is ~0.03% (weight fraction is 0.0%). It means the fit to the rock-salt phase is below the detection limit of our XRD measurement. It also means on average this is a very limited amount of disordered rock-salt formation in our VG-NMC811 materials.

Oxygen deficiency in layered oxides can originate from many factors such as high temperature treatment, annealing under a reduced O_2_ pressure or long calcination time^27–31^. We will discuss how to achieve the oxygen off-stoichiometry while preserving a near-perfect O3 lattice in the “Synthesis” section below.

The same STEM-EELS and STEM-EDS analyses are also performed on the conventional NMC811 material (Supplementary Fig. 7). In contrast to the VG-NMC811’s results, the spatially resolved Ni L_2,3_ edges show no chemical shifts indicating that the Ni valence state is constant from the surface to the center of secondary particles (Supplementary Fig. 7b and c). The Mn and Co L_2,3_ edges have no changes indicating Mn and Co’s valence states are also constant over the whole particle (Supplementary Fig. 7d). These results are cross validated by the pre-peak feature of the oxygen K edge (Supplementary Fig. 7d). The oxygen K edge’s pre-peak reflects the strength of O_2*p*_-TM_3*d*_ hybridization. Its intensity tracks the population of the transition metal *d*-holes. The fact that the O K pre-peaks’ position and intensity remain unchanged indicates the transitional metals’ bonding with oxygen remains constant throughout the particle. It again confirms Ni, Mn, and Co’s oxidation states are constant throughout the secondary particle. The STEM-EDS quantification result shows that Ni/Mn/Co composition is constant throughout the whole particle (Supplementary Fig. 7e).

### Degradation mechanism and structural stabilization of VG-NMC811

**Fig. 2:**
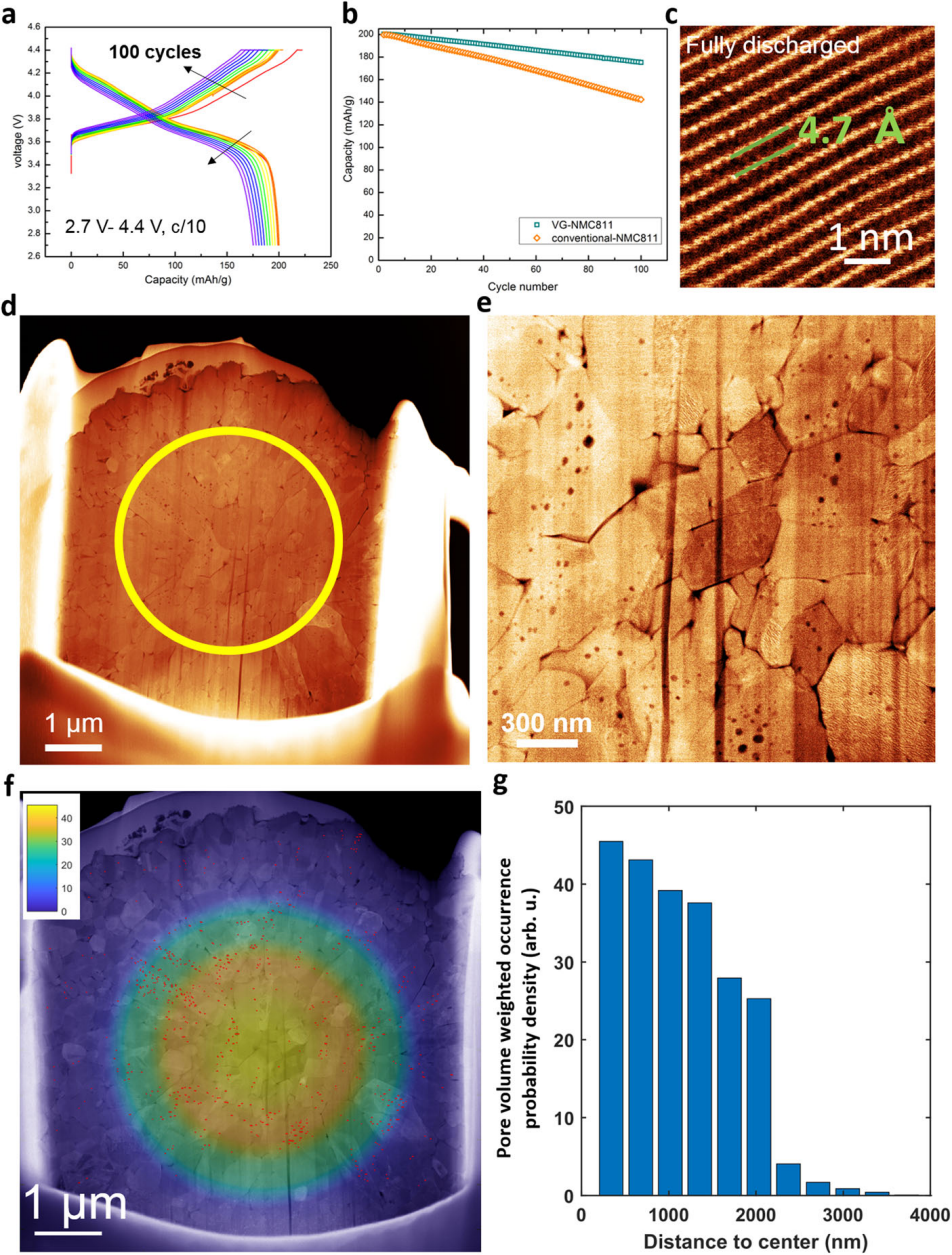
Electrochemical characterization of valence gradient NMC811 material (VG-NMC811). **a** Charge/discharge curves of VG-NMC811//lithium half-cell with cut-off voltage of 2.7 and 4.4 V. **b** Comparison of charge capacity for 100 cycles between VG-NMC811 half-cell and conventional NMC811 half-cell. HAADF-STEM images of VG-811 NMC after 40 electrochemical cycles. **c** Atomic-resolution Z-contrast image shows well-retained layered structure with 4.7 Å lattice spacing between two transition metal layers. **d** Cross-sectional image of secondary particle showing higher density of nanopores in the central area. **e** Magnified image of the central area of cycled secondary particle showing electrochemical cycling-induced nanopores. **f** The cross-sectional image with pore labels (red marks) and symmetrized pore volume density map overlaid. **g** The radially averaged pore volume weighted occurrence probability (see “Methods” for details).

The electrochemical performances of VG-NMC811 materials were tested in lithium half-cells in standard 2032 coin cells and the cycled electrodes from these cells were collected for the subsequent studies using electron microscopy, STEM-EELS, synchrotron-based X-ray imaging and spectroscopy. The half-cell was pre-cycled between 2.7 and 4.4 V at a charge/discharge rate of C/10 for 100 cycles using a 1 M LiPF6/EC/EMC electrolyte (Fig. 2a, see “Methods” for details). After 100 cycles, the specific charge/discharge capacity is retained over 175 mAh/g (Fig. 2b), showing that the capacity retention of VG-NMC811 is greatly improved over conventional NMC811 materials (142 mAh/g after 100 cycles). These results indicate that the main structures of the secondary particle and the crystal lattice of the layered transition oxide are well maintained. High-angle annular dark-field scanning transmission electron microscopy (HAAD-STEM) images of a VG-811 primary particle after 40 cycles (C/10 charge/discharge rate for the first three cycles and C/3 charge/discharge rate for the following 37 cycles) are shown in Fig. 2c. Since HAADF-STEM uses high-angle scattered electrons to form images, the contrast of the resulting image is sensitive to the projected atomic mass of the underlying atomic columns, which is usually referred to “Z-contrast”. The projection image in Fig. 2c was taken along [0, 1, 0] zone axis. A perfect layered structure cathode material is shown in Fig. 2c with alternation (4.7 angstrom repeating period) of a transition metal layer (bright) and a lithium layer (dark).

Very interestingly, we found that after electrochemical cycling, the density of nanopores is much higher in the center of the secondary particle (marked in yellow circle in Fig. 2d) than in the near-surface region. Figure 2e shows the nanopores in the central area of the secondary particle with larger magnification. To quantify the probability of pore formation over the entire particle, we hand labeled all pore locations and diameters (red bar) as shown in Fig. 2f. We then calculated the radially averaged pore occurrence probability weighted by pore volume and color coded the results to provide a direct visualization of nanopore density across the cycled secondary particle. The volume weighted nanopore density as a function of distance to the center is shown in the bar plot in Fig. 2g (see “Methods” for details). These results clearly show that the chance of forming more pores with larger volumes is statistically lower in the area closer to the surface. The generation of nanopores is closely correlated to the irreversible oxygen loss and propagation of oxygen vacancies, which usually associated with phase transformation, cation intermixing, as well as micro/nanocracks formation during charge/discharge cycling^7,32,33^. Analysis of the pore distribution of another particle is provided in Supplementary Fig. 8 and it shows a similar trend. During electrochemical charging process, Ni^3+^ can be oxidized to Ni^4+^, which is not chemically stable and tends to be reduced to Ni^2+^. To compensate the charge difference between Ni^4+^ and Ni^2+^, oxygen is released from the crystal lattice and leave oxygen vacancies behind, which can aggregate and form nanopores in the primary particles as shown in Fig. 2e. Typically, such nanopores form preferably at or near the surface of the secondary particle due to the cathode-electrolyte interaction and surface structure instability. The fact that there are more pores in the bulk center of this VG-NMC811 material indicates that the Ni^2+^ at the surface and Ni-valence gradient in the near-surface region indeed suppress the structure degradation by protecting the surface and near-surface region from undesired nanopore formation. Since the valence state of Ni at surface and near-surface region of the secondary particle of VG-NMC811 is at or close to the low valence state of Ni^2+^, the structure in this region is more stable against oxygen loss and degradation. This finding supports our hypothesis—the key factor responsible for the improved stability in many concentration-gradient cathode materials might be the reduced Ni-valence state at and near the surface, rather than the reduced Ni concentration. Specifically, in this VG-NMC811 material, it is the low Ni-valence state at and near the surface of the particles that suppresses the oxygen release and improves the capacity retention. In addition, although at a much lower occurrence, the pristine particles do present a few nanopore (Supplementary Fig. 9) which may serve as nuclei as previously found by Janek, Volz, and coauthors^34^.

**Fig. 3:**
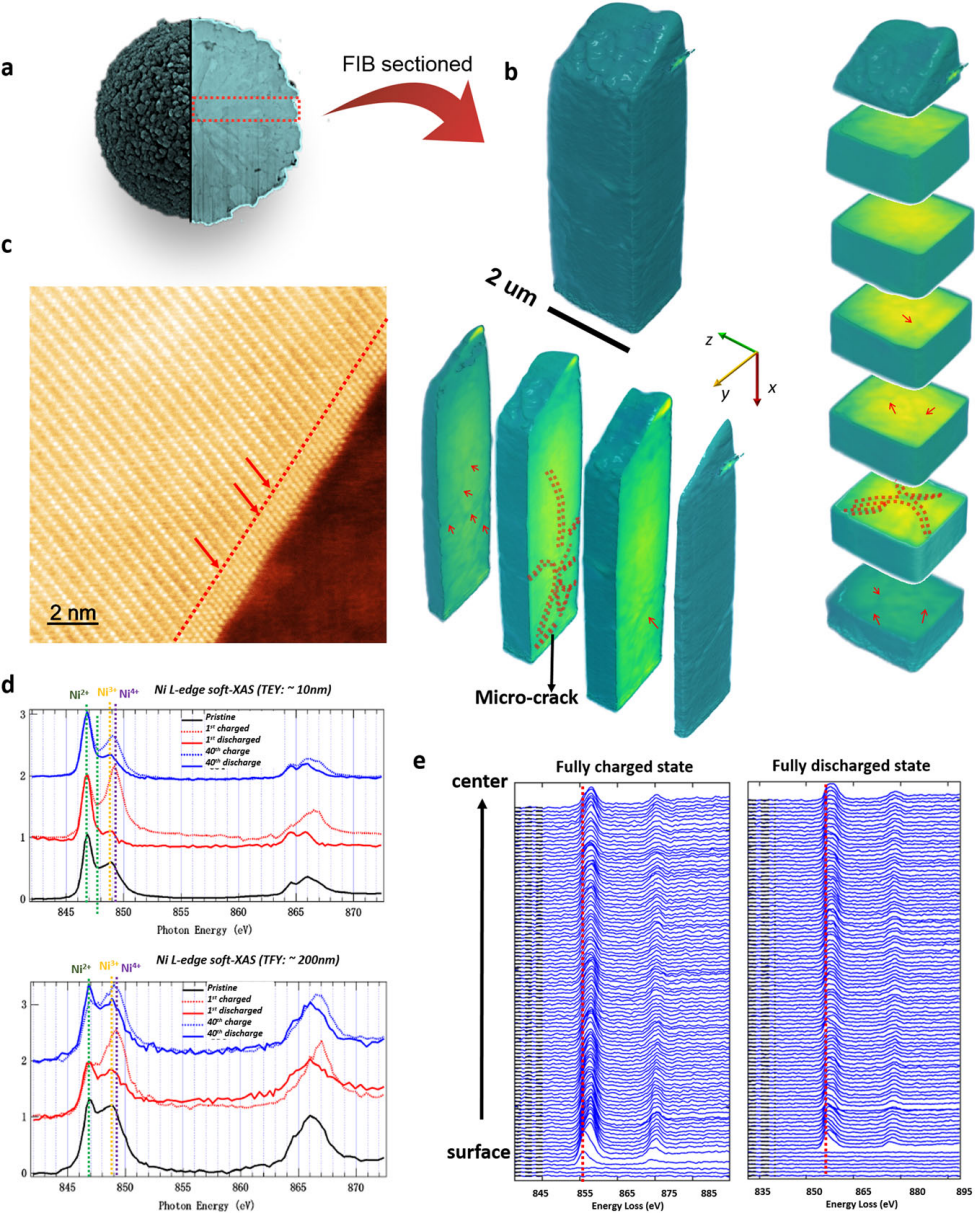
Multi-scale length structure and chemical evolution of the valence gradient NMC811 material (VG-NMC811) after 40 electrochemical cycling. **a** Schematics of where the pillar was cut from the cycled secondary particle. **b** Three-dimensional X-ray tomography of the central pillar of a cycled secondary particle as marked in (**a**), which shows the formation of micro-cracks. **c** Atomic-scale HAAD-STEM imaging of the surface of an internal primary particle showing surface rock-salt reconstruction. **d** Soft X-ray absorption spectroscopy (XAS) of Ni L-edge for materials in pristine, after 1st cycle and after 40th cycle collected in total electron yield (TEY) and total fluorescence yield (TFY) modes. **e** The electron energy loss spectra of Ni L_2,3_-edge from the surface to the center at charged state (4.4 V) and discharged state (2.7 V).

To understand the evolution of microstructure of the secondary particle and its effect on the oxygen loss in the bulk center of the secondary particle, we performed nanotomography imaging of cycled VG-NMC811 samples using a ptychographic tomography technique with a hard X-ray nanoprobe. To obtain high-resolution imaging 3D imaging, we used a dual-beam focused ion beam instrument to cut out a pillar-shaped piece of the material along a central radial direction of the secondary particle as the red rectangle shows in Fig. 3a. The phase-contrast images recovered by ptychography at all projection angles were sequentially reconstructed by tomography to give a 3D structure with a 20 nm voxel size. Figure 3b shows three representative cross-section images of the 3D tomographic reconstruction of the central part of a cycled secondary particle along two different directions. The results show micro-cracks between primary particles extend from the secondary particle’s center to the outer area of the particle. Around those cracks, we found that the primary particle surface transformed into a rock-salt structure (Fig. 3c). This degradation is likely attributed to electrolyte exposure which suggests these micro-cracks provide a pathway for electrolyte penetration and result in rapid oxygen loss. These micro-cracks can also lead to poor electronic conductivity between the primary particles, which could also contribute to the capacity decay in VG-NMC811 materials as shown in Fig. 2b. Based on our observation at both the secondary particle and the primary particle level, it is highly possible that the degradation mechanism in this VG-NMC811 material is dominated by oxygen loss from the central part of the secondary particles that has higher Ni-valence state as well as pathways to electrolyte penetration.

To probe whether the Ni-valence gradient is still retained after charge/discharge cycles, we performed STEM-EELS mapping from the secondary particle surface to the center along the radial direction on a discharged particle (2.7 V) and a charged particle (4.4 V) after 50 cycles. The EELS results in Fig. 3e show in both charged and discharged states, the valence gradient is preserved. In the charged state, the valence state of Ni at the surface is at Ni^2+^ and in near-surface region is between Ni^2+^ and Ni^4+^, while the Ni^3+^ observed in pristine sample in the center bulk is oxidized to Ni^4+^ when lithium is extracted, indicated by the larger shift to the higher energy of Ni L_3_ edge comparing with the spectra of the discharged sample. Since the electron beam only probe a very small spot of the sample and may have the statistical reliability issue, we also performed soft X-ray absorption spectroscopy (XAS) to obtain the average information about the Ni-valence state collected from large part of the sample with higher energy resolution. Moreover, the XAS spectra collected through the total electron yield (TEY) and total fluorescence yield (TFY) channel provide the surface and bulk sensitive information, respectively. Figure 3d displays the XAS spectra of a series of representative electrodes with the general feature assignments indicated by the vertical dotted lines^35^. In the pristine state, Ni^2+^ feature is more dominant in the TEY spectrum while Ni^3+^ is more dominant in the TFY spectrum, implying that more Ni^2+^ in the surface/near-surface region. This result is consistent with our EELS result in Fig. 1b for pristine materials. Similarly, at the charged sate, Ni^2+^ still dominates at and near-surface region, while most of Ni is oxidized to Ni^4+^ in the bulk^36^. The soft X-ray spectroscopy results (dashed line curves in Fig. 3d) also indicate a significant portion of Ni^2+^ ions in the near-surface region also participate in the redox reaction during the charge/discharge process suggesting that those Ni^2+^ ions are electrochemically active, not in the form of NiO rock-salt structure. In another word, the Ni^2+^ ions at the top surface (about 2–3-nm depth) are in the NiO rock-salt structure and electrochemical inactive, while the Ni^2+^ rich region near surface (about 900-nm depth) still contributes to the capacity while stabilizing the secondary particle structure and suppressing the formation of structure degradation initiators such as nanopores and micro/nanocracks.

### Improved thermal stability of VG-NMC811

Thermal stability of cathode materials is very important for the safety characteristics of Li-ion batteries. To address the thermal stability of VG-NMC811, in situ time-resolved X-ray diffraction coupled with mass spectroscopy (TR-XRD/MS) studies during heating were carried out. It is worthwhile to point out that the structure degradation can be accelerated during heating. Therefore, the structural change information obtained through thermal abuse study can also provide valuable reference mimicking the condition of extensive electrochemical cycling in a short time, which is quite useful when doing in situ studies with very limited synchrotron beam time.

**Fig. 4:**
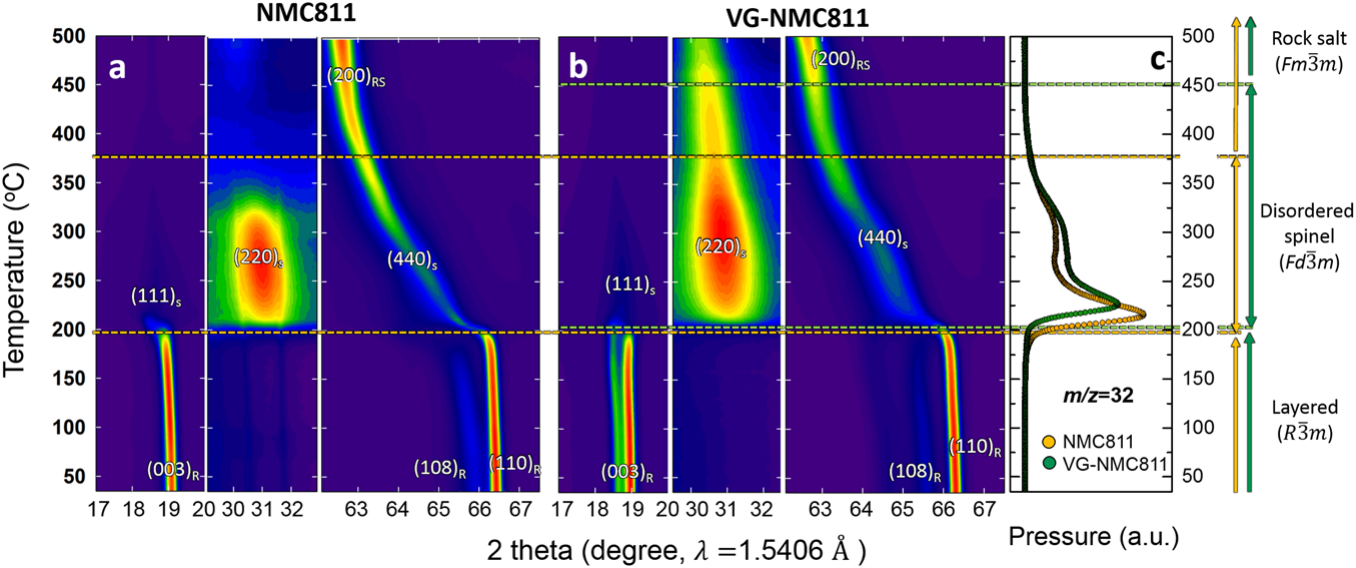
Time-resolved X-ray diffraction measurements of the cathode materials during thermal abuse tests. Contour plot of the time-resolved X-ray diffraction (TR-XRD) patterns at the selected 2*θ* range of the **a** charged conventional NMC811 and **b** the valence gradient NMC811 material (VG-NMC811) during in situ heating to 500 °C. **c** O_2_ released from conventional NMC811 and VG-NMC811 during in situ heating to 500 °C.

For comparison, both VG-NMC811 and conventional NMC811 material were studied. They were cycled in 2032 coin cells between 2.7 and 4.4 V for 49 cycles and then charged to 4.4 V at the 50th cycle. Constant current (i.e., galvanostatic) at C/10 rate for the first three cycles and C/3 rate for the following 47 cycles were applied to the coin cells. Contour plot of the TR-XRD patterns at the selected 2*θ* ranges for the conventional NMC811 and VG-NMC811 during heating to 500 °C are shown in Fig. 4a and b, respectively (at a heating rate of 2 °C/min). Both of the two samples showed that at charged state (4.4 V) after 50 cycles, the crystal structures are preserved in layered structure of rhombohedral symmetry (*R*$\bar{3}$*m*). During in situ heating, both materials first showed structure evolution from layered structure to disordered spinel (LiMn_2_O_4_-type, *Fd*$\bar{3}$*m*) as a result of thermal decomposition. The appearing of (220)_s_ peaks and disappearing of (003)_R_ peak with the merging of (108)_R_/(110)_R_ peak indicate the beginning of phase transformation from a layered structure to a disordered spinel structure, which accompanying with oxygen loss (Fig. 4c) during the heating^7^. However, the onset temperature of first phase transformation and further structure evolutions of these two samples are quite different. As is shown in Fig. 4a, b, the temperature of first thermal decomposition and phase transformation from the layered to disordered spinel phase and accompanied oxygen release for VG-NMC811 (*ca*. 205 °C) is slightly higher than conventional NMC811(*ca*. 195 °C). Moreover, the second-phase transformation from the disordered spinel to rock-salt structure ended at *ca*. 375 °C for conventional NMC811, which is indicated by the disappearing (220)_s_ peak and growth of (200)_RS_ peak. In contrast, the second phase transition of charged VG-NMC811 material is delayed to much higher temperature and the disorder spinel structure is retained up to 450 °C, showing that oxygen release and structure change in VG-NMC811 are significantly suppressed. These results suggest that VG-NMC811 has much better thermal stability, which can be attributed to the Ni-valence gradient in the subsurface region of the secondary particles.

### Synthesis

In studying the degradation mechanism and searching the strategy to stabilize the structure of high-Ni-content NMC materials, most past research had focused on creating a content distribution of transition metal elements, especially the Ni content, by introducing doping elements or a Ni concentration gradient^37^. In this paper, we demonstrate that the valence state of Ni is another important and maybe more dominant factor, which is closely related to the stoichiometry of the oxygen anion, and should be studied more thoroughly. Since it is designed to keep the Ni–Mn–Co composition constant throughout the VG-NMC811 particle, the introduction of lower Ni-valence states can be achieved through oxygen off-stoichiometry that we discussed in the previous sections. In practice, synthesizing layered cathode materials with both oxygen off-stoichiometry and a good layered structure is not easy, because reducing oxygen partial pressure to reduce the Ni-valence state would result in anti-site defect (i.e., transition metal occupancy in the lithium layer) which reduces the capacity^38,39^. Particularly, with a reduced oxygen partial pressure or oxygen flow rate, it will cause the interior of the secondary particle to be more oxygen-deficient because oxygen needs to diffuse through the extra length to reach the interior. In order to deal with this problem, a special synthesis procedure was designed. According to the Ellingham diagram^40^, Mn binds to oxygen much stronger than Ni and Co; therefore, we can use some extra Mn on the top surface of the precursor to generate a Mn-concentration gradient. As the extra Mn diffuses inward during calcination, it helps to enhance oxygen diffusion from the surface to the center of the particle. In particular, during the Mn diffusion/homogenization process, Mn moves with oxygen (Mn–O) inward whereas the Ni moves outward without oxygen (Ni-□_O_) causing the valence gradient to form. Although we start with a Mn-concentration-gradient precursor particle, with Mn diffusion over sufficient time under an oxygen-deficient calcination environment, we were able to obtain composition-homogenized NMC811 materials with the desired Ni-valence gradient as designed. Guided by this design principal, we deliberately synthesized the hydroxide precursor with a Ni-rich core and Mn-rich shell structure by using a coprecipitation method (see “Methods” for details). As shown by the X-ray nanobeam 2D XRF mapping (Supplementary Fig. 10), the as-synthesized hydroxide precursor particle has a Mn-rich surface and a Ni-rich core. Then, Ni/Mn/Co hydroxide precursor is mixed with Li(OH) and calcinated in oxygen gas flow and Ni-valence gradient NMC811 materials as designed was successfully synthesized (see “Methods” for details).

To have a more in-depth understanding of the synthesis parameter, we analyzed NMC811 materials calcinated in different oxygen partial pressure conditions (pure oxygen and air) with the same core–shell Ni/Mn/Co hydroxide precursors shown in Supplementary Fig. 10a and same heating profile (see “Methods”). As shown in the XRD results in Supplementary Figs. 11 and 12 and Supplementary Tables 3 and 4, NMC811 materials synthesized in pure oxygen (100% O_2_) have less cation intermixing than the ones calcinated in the air (21% O_2_), which is indicated by the peak intensity ratio of (003)/(104) (Supplementary Fig. 13). This result implies that a relatively oxygen-deficient calcination environment (i.e., in the air) leads to a higher level of cation intermixing. The enrichment of oxygen (i.e., flowing pure oxygen) in the calcination process is one of the most important synthesis conditions that mitigate the reduction of Ni^3+^ to Ni^2+^, thus avoid large amount of cation intermixing, as appeared in related studies of high-Ni-content oxide materials^11,41,42^. Supplementary Fig. 10b and c shows X-ray fluorescence mapping of NMC811 materials synthesized in pure oxygen and air. It is also found that after 20 h of calcination at 780 °C in both oxygen and air, the compositional core–shell structure almost disappears and the elemental distribution becomes uniform in agreement with our aforementioned design principle. Here, the elemental redistribution and diffusion of transition metal during calcination are very likely driven by the homogenization of the chemical potentials. There is slight surface Mn segregation in NMC811 calcinated in pure oxygen. This absorbate induced segregation is also due to the strong association between Mn and O. (Many previous studies have shown the less noble metal in an alloy/solid solution would segregate to the surface under the oxidation conditions^43,44^.) On the other hand, Mn diffuses even more uniformly in NMC811 calcinated in air where oxygen is more deficient than in pure oxygen gas. Our work shows that given sufficiently long calcination time we can utilized the stronger Mn–O bond strength to carry a sufficient amount of oxygen into the core area of the secondary particle whileing keep the Ni cations at the Ni^+2^ state at the surface. This synthesis design principal can be directly applied to large-scale manufacturing of cathode powders where oxygen is deficient due to the limited oxygen mass transport in large batches of materials.

## Discussion

By combining synchrotron-based X-ray imaging and spectroscopy techniques with high-resolution electron microscopy and spectroscopy, we carried out a multi-scale investigation of the Ni-valence-gradient effect in high-Ni-content cathode materials. Structure and spatially resolved chemical information from the atomic level to the micron level is combined to rationalize the degradation mechanism in Ni-based layered cathode material. Irreversible structure degradation such as micro-cracks and nanopores caused by charge–discharge cycling are observed at both the primary particle and secondary particle level, which are correlated with less capacity fading in VG-NMC811 than the conventional NMC811 cathode materials. The effect of Ni-valence state on structure stabilization and electrochemical performances is reported, providing an approach and valuable guidance for future cathode material design. Our results suggest that introducing Ni-valence gradient from the surface to center of the secondary particle of high-nickel-content material can effectively stabilize the surface of secondary particles and mitigate the electrochemical performance fading caused by internal structure degradation without sacrificing the capacity. Also, it is found that oxygen partial pressure during calcination can be the key tuning parameter that determines the hierarchical structure of high-Ni-content cathode materials; and Mn-concentration gradient can effectively solve the problem of oxygen non-stoichiometry during calcination process especially for large-scale synthesis.

## Methods

### Synthesis of valence gradient LiNi_0.8_Mn_0.1_Co_0.1_O_2_

The synthesis of valence gradient LiNi_0.8_Mn_0.1_Co_0.1_O_2_ is based on the method previously reported in ref. ^45^. We prepared the hydroxide precursor that consists of Ni_0.90_Co_0.05_Mn_0.05_(OH)_2_ in the core portion of the particle and Ni_1/3_Mn_1/3_Co_1/3_(OH)_2_ in the surface volume of the particle in a 20-liter batch reactor. An aqueous solution (NiSO_4_·6H_2_O, CoSO_4_·7H_2_O, and MnSO_4_·5H_2_O) for forming the core composition of Ni:Co:Mn = 90:5:5 was prepared in the first solution tank. It was pumped into the reactor, which was pre-filled with ammonium hydroxide solution, deionized water, and sodium hydroxide solution and the reactor was protected by a nitrogen atmosphere. At the same time, a 10 mol L^−1^ sodium hydroxide solution was pumped into the reactor for pH adjustment and a 3.8 mol L^−1^ ammonium hydroxide solution was pumped into the reactor for the chelating purpose. For forming the surface composition of Ni:Co:Mn, 1:1:1, the NiSO_4_·6H_2_O, CoSO_4_·7H_2_O, and MnSO_4_·5H_2_O solution was prepared in a second solution tank and it was pumped into the first solution tank when the hydroxide precursor particles, that have a composition of Ni_0.90_Co_0.05_Mn_0.05_(OH)_2_, were grown to the prescribed particle size. By doing so, the composition of the feed solution of the first metal solution tank gradually changes of Ni:Co:Mn molar ratio from 90:5:5 to 1:1:1 and a feed solution of the composition thus changed is deposited on the precursor particle core (Ni_0.90_Co_0.05_Mn_0.05_(OH)_2_). The precursors for the conventional NMC811 material were synthesized by a one-step coprecipitation process with no core–shell structure. After the coprecipitation process, the produced core-gradient-structured hydroxide precursors from the batch reactor were filtered, washed, and dried for 20 h at 100 °C. In the next step, the dried precursor was mixed with LiOH·H_2_O and calcined at 800 °C for 10 h under oxygen flow.

### Electrochemical measurement

For cathode electrode fabrication, the synthesized core-gradient-structured NMC811 powder, Super P carbon, and 8 wt% polyvinylidene fluoride (PVDF) binder solution dissolved in N-methyl-2-pyrrolidone (NMP) (90:5:5) were mixed. The prepared slurry was spread onto Al foil current collector by using the doctor blade deposition method and then dried in an oven at 80 °C under vacuum. Coin-cell tests were performed using 2032 with cathode loadings around 5 mg cm^−2^ and pure lithium metal as the anode under a flooded-electrolyte condition. The electrolyte solution was 1 M LiPF_6_ in a mixture of ethylene carbonate (EC) and ethyl methyl carbonate (EMC), 3:7 volume ratio, and the separator is Celgard 2325. The fabrication of the half coin-cell in an Argon-filled glove box. Assembled coin cells rested at OCV (3.0 V) for 10 h and were tested by the Maccor (Series 4000) for cycle ability test with a current density at 0.1 C rate (20 mA g^−1^), the voltage range in 2.7–4.4 V for 100 cycles at 30 °C.

### TEM imaging and spectroscopy

The TEM samples were prepared by FIB directly from dissembled cathode electrodes without rinsing. The atomic-resolution scanning transmission electron microscopy (STEM) imaging of the VG-NMC811 cathode material was performed on a FEI Talos F200X (200 keV, probe forming semi-angle = 10 mrad) in a high-angle annular dark-field mode. The spatially resolved electron energy loss spectra were collected in the STEM mode by an Gatan Enfinia spectrometer with dispersions of 0.1 eV/channel and 0.25 eV/channel.

### Symmetrized pore volume density map

To extract the pore density map, we hand label all pore locations and diameters in the image shown in Fig. 2f. We then calculated the radially averaged pore occurrence probability weighted by pore volume using Eq. (1). This extracted quantity not only reflects the radially averaged occurrence probability but also the pore volume information. A combination of both reflect how much oxygen is released per unit area as function of radial distance.

1 $${{\mathrm{Prob}}}_{v}(r)=\mathop{\sum }\limits_{{r}_{i}\ge r}^{{r}_{i} < r+{dr}}\frac{32/3\pi {D}_{i}{\left({r}_{i}\right)}^{3}}{2\pi {rdr}}$$

where *D*_*i*_ is the diameter of pore *i* that falls within the area bounded by radius *r* and *r* + *dr*.

### FIB sample preparation

An in situ lift-out method was used to prepare the TEM cross-sectional samples on the Helios Nanolab 600 dualbeam FIB with a final Ga^+^ milling step performed at 5 keV. The TEM samples of the cathode particles were lifted out directly from pristine electrodes or dissembled cathode electrodes without rinsing.

### Nanobeam X-ray imaging

X-ray imaging was performed at beamline 3-ID (HXN) of the National Synchrotron Light Source II at Brookhaven National Laboratory^46^. The X-ray imaging experiment was carried out at 10 keV by focusing the coherent monochromatic X-rays using a Fresnel X-ray zone plate. Ptychographic tomography measurements were performed by collecting a total of 86 projections from −86 to 84°, with 2° intervals. At each projection angle, a 2D on-the-fly raster scan was conducted with a dwell time of 50 ms and a 30 nm step size. The phase-contrast image at each angle was recovered by a GPU paralyzed ptychographic reconstruction engine^47^ using 500 iterations of the difference map algorithm. The tomographic reconstruction was carried out with TomoPy^48^ using 100 interactions of the ordered-subset penalized maximum likelihood algorithm with weighted linear and quadratic penalties. 3D visualization of the reconstructed data was carried out using the Tomviz software^49^.

Two-dimensional fluorescence imaging was performed using a step size of 30 nm/pixel and a dwell time of 50 ms. The obtained fluorescence spectra were fitted using the PyXRF software^50^ to produce elemental distribution images.

### Full-field transmission X-ray microscopy (TXM) imaging

Transmission X-ray microscopy imaging of the cathode materials was performed at the Full Field X-ray Imaging beamline (18-ID FXI) at NSLSII. The X-ray microscopy at the FXI beamline offers a resolution of 30 nm. To prepare the samples for TXM imaging. The particles were directly scraped off the electrode surface (without rinsing) in the glove box and loaded into a Kapton tube. The Kapton tubes were sealed with epoxy in the glove box before transferred to the FXI beamline.

### X-ray diffraction

In situ TR-XRD data were collected at beamline 17-BM-B (*λ* = 0.45220 Å) of the Advanced Photon Source (APS) at Argonne National Laboratory. The details of the sample preparation and experimental setup have been published previously^51^. TR-XRD patterns (~1 min for each XRD scan) and MS signal were simultaneously collected continuously as the sample was heated from room temperature to 500 °C for 2 h (i.e., at a heating rate of ~4 °C min^−1^). For easy comparison with results in the literature, the 2*θ* angles have been converted to values corresponding to the Cu Kα radiation (*λ* = 1.5406 Å).

Ex situ XRD was collected at beamline 17-BM-B (*λ* = 0.45118 Å) of APS and beamline 28-ID-2 (*λ* = 0.1949 Å) of NSLSII at Brookhaven National laboratory. The two-dimensional diffraction images were integrated and converted to 1D diffraction patterns using GSAS II^52^ and Fit2d^53^. Structural refinements were performed by the Rietveld method using GASA II.

### Soft X-ray absorption spectroscopy (sXAS)

Ni, Co, and Mn L_2,3_-edge soft XAS measurements were performed in iRIXS end station at beamline 8.0.1 of the advanced Light Source (ALS) at Lawrence Berkeley National Lab^54^. The undulator and spherical grating monochromator supply a linearly polarized photon beam with resolving power up to 6000. The experimental energy resolution is about 0.15 eV without considering the core hole broadening. Experiments were performed at room temperature and with the incident beam linearly polarized. All the spectra were normalized to the beam flux measured by the upstream gold mesh. For the measurement, 2032 coin cells were cycled with different cycle numbers. Afterward, they were disassembled carefully in Ar filled glove box, then the cathodes collected were immediately rinsed with EMC. The cathodes were cut and mounted onto sample holders in Ar glove box, then transferred into the XAS vacuum chamber through a home-designed sample transfer kit that is sealed in Ar glove box to avoid any air exposure.

## Supplementary information

Supplementary Information

## Data Availability

The authors declare that the data supporting the findings of this study are available within the paper and its Supplementary Information files.

## References

[1] Lee K-S, Myung S-T, Amine K, Yashiro H, Sun Y-K (2007). Structural and electrochemical properties of layered Li [Ni_1−2x_Co_x_Mn_x_]O_2_ (x = 0.1–0.3) positive electrode materials for Li-ion batteries. J. Electrochem. Soc..

[2] Sun Y-K (2007). Physical and electrochemical properties of Li [Ni_0.4_Co_x_Mn_0.6−x_]O_2_ (x = 0.1–0.4) electrode materials synthesized via coprecipitation. J. Electrochem. Soc..

[3] Sung-Kyun J (2014). Understanding the degradation mechanisms of LiNi_0.5_Co_0.2_Mn_0.3_O_2_ cathode material in lithium ion batteries. Adv. Energy Mater..

[4] Yu H (2014). Study of the lithium/nickel ions exchange in the layered LiNi_0.42_Mn_0.42_Co_0.16_O_2_ cathode material for lithium ion batteries: experimental and first-principles calculations. Energy Environ. Sci..

[5] MD J, Christian P, WJ G, AD P, Javier B (2013). Observation of microstructural evolution in li battery cathode oxide particles by in situ electron microscopy. Adv. Energy Mater..

[6] Hwang S, Kim DH, Chung KY, Chang W (2014). Understanding local degradation of cycled Ni-rich cathode materials at high operating temperature for Li-ion batteries. Appl. Phys. Lett..

[7] Bak S-M (2013). Correlating structural changes and gas evolution during the thermal decomposition of charged Li_x_Ni_0.8_Co_0.15_Al_0.05_O_2_ cathode materials. Chem. Mater..

[8] Mu L (2018). Oxygen release induced chemomechanical breakdown of layered cathode materials. Nano Lett..

[9] Noh H-J, Youn S, Yoon CS, Sun Y-K (2013). Comparison of the structural and electrochemical properties of layered Li[Ni_x_Co_y_Mn_z_]O_2_ (x = 1/3, 0.5, 0.6, 0.7, 0.8 and 0.85) cathode material for lithium-ion batteries. J. Power Sources.

[10] Ryu H-H, Park K-J, Yoon CS, Sun Y-K (2018). Capacity fading of Ni-rich Li[Ni_x_Co_y_Mn_1–x–y_]O_2_ (0.6 ≤ x ≤ 0.95) cathodes for high-energy-density lithium-ion batteries: bulk or surface degradation?. Chem. Mater..

[11] Ohzuku T, Ueda A, Nagayama M (1993). Electrochemistry and structural chemistry of LiNiO_2_ (R3̅m) for 4 volt secondary lithium cells. J. Electrochem. Soc..

[12] Thomas MGSR, David WIF, Goodenough JB, Groves P (1985). Synthesis and structural characterization of the normal spinel Li[Ni_2_]O_4_. Mater. Res. Bull..

[13] GW E (2016). Persistent state-of-charge heterogeneity in relaxed, partially charged Li_1−x_Ni_1/3_Co_1/3_Mn_1/3_O_2_ secondary particles. Adv. Mater..

[14] Mao Y (2019). High-voltage charging-induced strain, heterogeneity, and micro-cracks in secondary particles of a nickel-rich layered cathode material. Adv. Funct. Mater..

[15] Yan P (2017). Intragranular cracking as a critical barrier for high-voltage usage of layer-structured cathode for lithium-ion batteries. Nat. Commun..

[16] Yan P (2018). Coupling of electrochemically triggered thermal and mechanical effects to aggravate failure in a layered cathode. Nat. Commun..

[17] Lin F (2014). Surface reconstruction and chemical evolution of stoichiometric layered cathode materials for lithium-ion batteries. Nat. Commun..

[18] Cho J, Kim T-J, Kim J, Noh M, Park B (2004). Synthesis, thermal, and electrochemical properties of AlPO_4_-coated LiNi_0.8_Co_0.1_Mn_0.1_O_2_ cathode materials for a Li-ion cell. J. Electrochem. Soc..

[19] Sun Y-K (2009). High-energy cathode material for long-life and safe lithium batteries. Nat. Mater..

[20] Sun Y-K (2012). Nanostructured high-energy cathode materials for advanced lithium batteries. Nat. Mater..

[21] Lin F (2016). Metal segregation in hierarchically structured cathode materials for high-energy lithium batteries. Nat. Energy.

[22] Sun YK (2011). A novel concentration-gradient Li[Ni_0.83_Co_0.07_Mn_0.10_]O_2_ cathode material for high-energy lithium-ion batteries. J. Mater. Chem..

[23] Yoon SJ (2014). Nanorod and nanoparticle shells in concentration gradient core-shell lithium oxides for rechargeable lithium batteries. Chemsuschem.

[24] Li Y (2016). Synthesis of full concentration gradient, cathode studied by high energy X-ray diffraction. Nano Energy.

[25] Cho Y, Oh P, Cho J (2013). A new type of protective surface layer for high-capacity Ni-based cathode materials: nanoscaled surface pillaring layer. Nano Lett..

[26] Liu H (2016). Spatially resolved surface valence gradient and structural transformation of lithium transition metal oxides in lithium-ion batteries. Phys. Chem. Chem. Phys..

[27] Levasseur S (2003). Oxygen vacancies and intermediate spin trivalent cobalt ions in lithium-overstoichiometric LiCoO_2_. Chem. Mater..

[28] Lim J-M (2016). Mechanism of oxygen vacancy on impeded phase transformation and electrochemical activation in inactive Li_2_MnO_3_. ChemElectroChem.

[29] Armstrong AR (2006). Demonstrating oxygen loss and associated structural reorganization in the lithium battery cathode Li[Ni_0.2_Li_0.2_Mn_0.6_]O_2_. J. Am. Chem. Soc..

[30] Xia Y (2007). Oxygen deficiency, a key factor in controlling the cycle performance of Mn-spinel cathode for lithium-ion batteries. J. Power Sources.

[31] Tang Z-K, Xue Y-F, Teobaldi G, Liu L-M (2020). The oxygen vacancy in Li-ion battery cathode materials. Nanoscale Horiz..

[32] Hu E (2018). Evolution of redox couples in Li-and Mn-rich cathode materials and mitigation of voltage fade by reducing oxygen release. Nat. Energy.

[33] Lin R (2019). Anomalous metal segregation in lithium-rich material provides design rules for stable cathode in lithium-ion battery. Nat. Commun..

[34] Ahmed S (2019). The role of intragranular nanopores in capacity fade of nickel-rich layered Li(Ni_1–x–y_Co_x_Mn_y_)O_2_ cathode materials. ACS Nano.

[35] Qiao R (2015). Direct experimental probe of the Ni(II)/Ni(III)/Ni(IV) redox evolution in LiNi_0.5_Mn_1.5_O_4_ electrodes. J. Phys. Chem. C..

[36] Yoon W-S (2005). Investigation of the charge compensation mechanism on the electrochemically Li-ion deintercalated Li_1-x_Co_1/3_Ni_1/3_Mn_1/3_O_2_ electrode system by combination of soft and hard X-ray absorption spectroscopy. J. Am. Chem. Soc..

[37] Manthiram A, Song B, Li W (2017). A perspective on nickel-rich layered oxide cathodes for lithium-ion batteries. Energy Storage Mater..

[38] Bi Y (2015). Correlation of oxygen non-stoichiometry to the instabilities and electrochemical performance of LiNi_0.8_Co_0.1_Mn_0.1_O_2_ utilized in lithium ion battery. J. Power Sources.

[39] Shim J-H (2014). Effects of heat-treatment atmosphere on electrochemical performances of Ni-rich mixed-metal oxide (LiNi_0.80_Co_0.15_Mn_0.05_O_2_) as a cathode material for lithium ion battery. Electrochim. Acta.

[40] Atkins, P. W. and De Paula, J. *Physical Chemistry: Thermodynamics and Kinetics* (Recording for the Blind & Dyslexic, 2007).

[41] Park SH (2002). The effects of oxygen flow rate and anion doping on the performance of the LiNiO_2_ electrode for lithium secondary batteries. Korean J. Chem. Eng..

[42] Guilmard M, Pouillerie C, Croguennec L, Delmas C (2003). Structural and electrochemical properties of LiNi_0.70_Co_0.15_Al_0.15_O_2_. Solid State Ion..

[43] Han L (2016). Interrogation of bimetallic particle oxidation in three dimensions at the nanoscale. Nat. Commun..

[44] Xia W (2018). Bimetallic nanoparticle oxidation in three dimensions by chemically sensitive electron tomography and in situ transmission electron microscopy. ACS Nano.

[45] Maeng S, Chung Y, Min S, Shin Y (2020). Enhanced mechanical strength and electrochemical performance of core–shell structured high–nickel cathode material. J. Power Sources.

[46] Nazaretski E (2017). Design and performance of an X-ray scanning microscope at the Hard X-ray Nanoprobe beamline of NSLS-II. J. Synchrotron Radiat..

[47] Dong, Z. et al. High-performance multi-mode ptychography reconstruction on distributed GPUs. in *2018 New York Scientific Data Summit (NYSDS)* (2018).

[48] Gürsoy D, De Carlo F, Xiao X, Jacobsen C (2014). TomoPy: a framework for the analysis of synchrotron tomographic data. J. synchrotron Radiat..

[49] Levin BD (2018). Tutorial on the visualization of volumetric data using *tomviz*. Microsc. Today.

[50] Li, L. et al. in *X-Ray Nanoimaging: Instruments and Methods III* (International Society for Optics and Photonics, 2017).

[51] Bak S-M (2014). Structural changes and thermal stability of charged LiNi_x_Mn_y_Co_z_O_2_ cathode materials studied by combined in situ time-resolved XRD and mass spectroscopy. ACS Appl. Mater. Interfaces.

[52] Toby BH, Von Dreele RB (2013). GSAS-II: the genesis of a modern open-source all purpose crystallography software package. J. Appl. Crystallogr..

[53] Hammersley A, Svensson S, Hanfland M, Fitch A, Hausermann D (1996). Two-dimensional detector software: from real detector to idealised image or two-theta scan. Int. J. High. Press. Res..

[54] Qiao R (2017). High-efficiency in situ resonant inelastic x-ray scattering (iRIXS) endstation at the Advanced Light Source. Rev. Sci. Instrum..

