# Supplementary material for: Hierarchical nickel valence gradient stabilizes high-nickel content layered cathode materials
Source: Nat Commun. 2021 Apr 20;12:2350. doi: 10.1038/s41467-021-22635-w (PMC8058063; doi:10.1038/s41467-021-22635-w)
Supplement: Supplementary file 1 — Supplementary Information [file 41467_2021_22635_MOESM1_ESM.pdf]

Supplementary Materials for

Hierarchical nickel valence gradient stabilizes high-nickel content layered cathode materials

Ruoqian Lin[1*#], Seongmin Bak[1*#%], Youngho Shin[2#], Rui Zhang[3], Chunyang Wang[3], Kim Kisslinger[4], Mingyuan Ge[5], Xiaojing Huang[5], Zulipiya Shadike[1], Ajith Pattammattel,[5] Hanfei Yan[5], Yong Chu[5], Jinpeng Wu[6], Wanli Yang[6], M. Stanley Whittingham[7], Huolin L. Xin[3*], Xiao-Qing Yang[1*]

1. Chemistry Division, Brookhaven National Laboratory, Upton, NY 11973, USA.
2. Applied Materials Division, Argonne National Laboratory, Lemont, IL 60439, USA.
3. Department of Physics and Astronomy, University of California, Irvine, CA 92697, USA.
4. Center for Functional Nanomaterials, Brookhaven National Laboratory, Upton, NY 11973, USA.
5. National Synchrotron Light Source II, Brookhaven National Laboratory, Upton, NY 11973, USA.
6 Advanced Light Source, Lawrence Berkeley National Laboratory, Berkeley, CA 94720, USA.
7. Materials Science & Engineering, Binghamton University, Binghamton, NY 13902, USA
# These authors contributed equally
% Current address: National Synchrotron Light Source II, Brookhaven National Laboratory, Upton, NY 11973, USA.
* Correspondences should be addressed to rulin@bnl.gov (R. L.) smbak@bnl.gov (S. B.) huolin.xin@uci.edu (H. L. X.) and xyang@bnl.gov (X.-Q. Y.)

Supplementary Table 1 XRD refinement results of pristine VG-811 calcined.

Space group: R-3m h							
a	b	c	alpha	beta	gamma	volume	
2.8746(1)	2.8746(1)	14.2082(8)	90	90	120	117.407	
label	element	multiplicity	x	y	z	fraction	Uiso
Li1	Li+	3	0	0	0	0.9703	0.0120
Li2	Li+	3	0	0	0.5	0.0297	0.0030
Ni1	Ni3+	3	0	0	0.5	0.7303	0.0079
Ni2	Ni2+	3	0	0	0	0.0297	0.0030
Co1	Co3+	3	0	0	0.5	0.12	0.0079
Mn1	Mn4+	3	0	0	0.5	0.12	0.0079

O1	O2-	6	0	0	0.24089	1	0.0194

Supplementary Table 2 Composition analysis of the three VG-NMC811particles shown in Supplementary Figure 5.

	**Ni (at. % ± 1 sigma %)**	**Co (at. % ± 1 sigma %)**	**Mn (at. % ± 1 sigma %)**
**Particle 1**			
**Surface**	78.32 ± 2.42	11.32 ± 0.40	10.35 ± 0.34
**Interior**	79.40 ± 2.45	11.42 ± 0.4	9.18 ± 0.31
**Particle 2**			
**Surface**	80.97 ± 2.49	10.95 ± 0.38	8.08 ± 0.27
**Interior**	81.78 ± 2.51	10.82 ± 0.37	7.41 ± 0.25
**Particle 3**			
**Surface**	77.99 ± 2.41	11.15 ± 0.39	10.86 ± 0.35
**Interior**	79.77 ± 2.44	11.74 ± 0.39	8.49 ± 0.28

Supplementary Table 3 XRD refinement results of precursors calcined in pure oxygen.

Space group: R-3m h							
a	b	c	alpha	beta	gamma	volume	
2.9069(0)	2.9069(0)	14.3788(0)	90	90	120	121.502	
label	element	multiplicity	x	y	z	fraction	Uiso
Li1	Li+	3	0	0	0	0.9821	0.0017
Li2	Li+	3	0	0	0.5	0.0179	0.003
Ni1	Ni3+	3	0	0	0.5	0.7421	0.0047
Ni2	Ni2+	3	0	0	0	0.0179	0.003
Co1	Co3+	3	0	0	0.5	0.12	0.0047
Mn1	Mn4+	3	0	0	0.5	0.12	0.0047
O1	O2-	6	0	0	0.24044	1	0.01095

Supplementary Table 4 XRD refinement results of precursors calcined in air.

Space group: R-3m h							
a	b	c	alpha	beta	gamma	volume	
2.9088(9)	2.9088(9)	14.3892(1)	90	90	120	121.749	
label	element	multiplicity	x	y	z	fraction	Uiso
Li1	Li+	3	0	0	0	0.9714	0.0019
Li2	Li+	3	0	0	0.5	0.0286	0.0030
Ni1	Ni3+	3	0	0	0.5	0.7314	0.0053
Ni2	Ni2+	3	0	0	0	0.0286	0.0030
Co1	Co3+	3	0	0	0.5	0.12	0.0053
Mn1	Mn4+	3	0	0	0.5	0.12	0.0053
O1	O2-	6	0	0	0.24050	1	0.01341
Li1	Li+	3	0	0	0	0.9703	0.0120

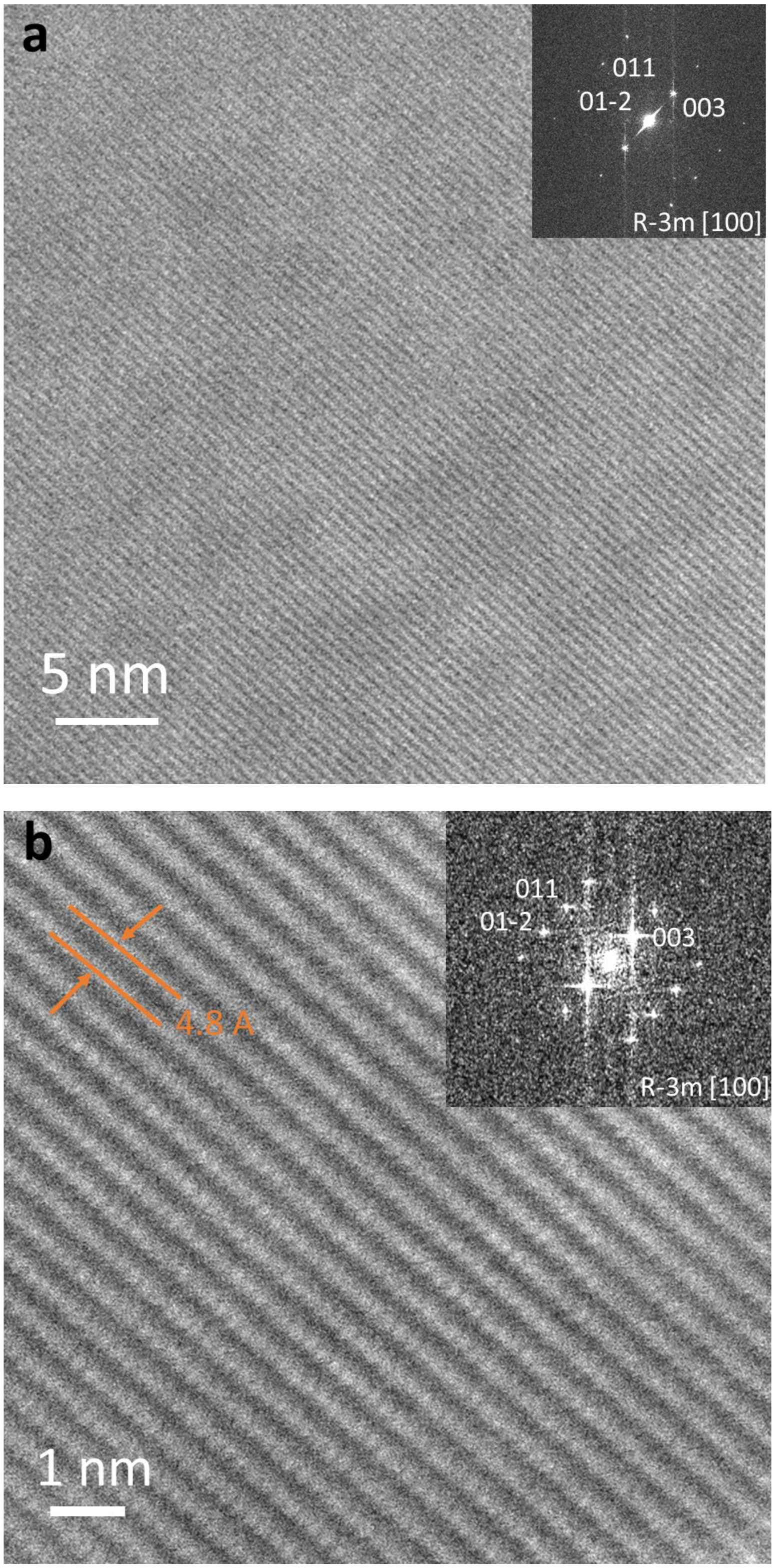

Supplementary Figure 1. Atomic-resolution HAADF-STEM images of pristine VG-NMC811 particles at the (a) lower magnification and the (b) higher magnification.

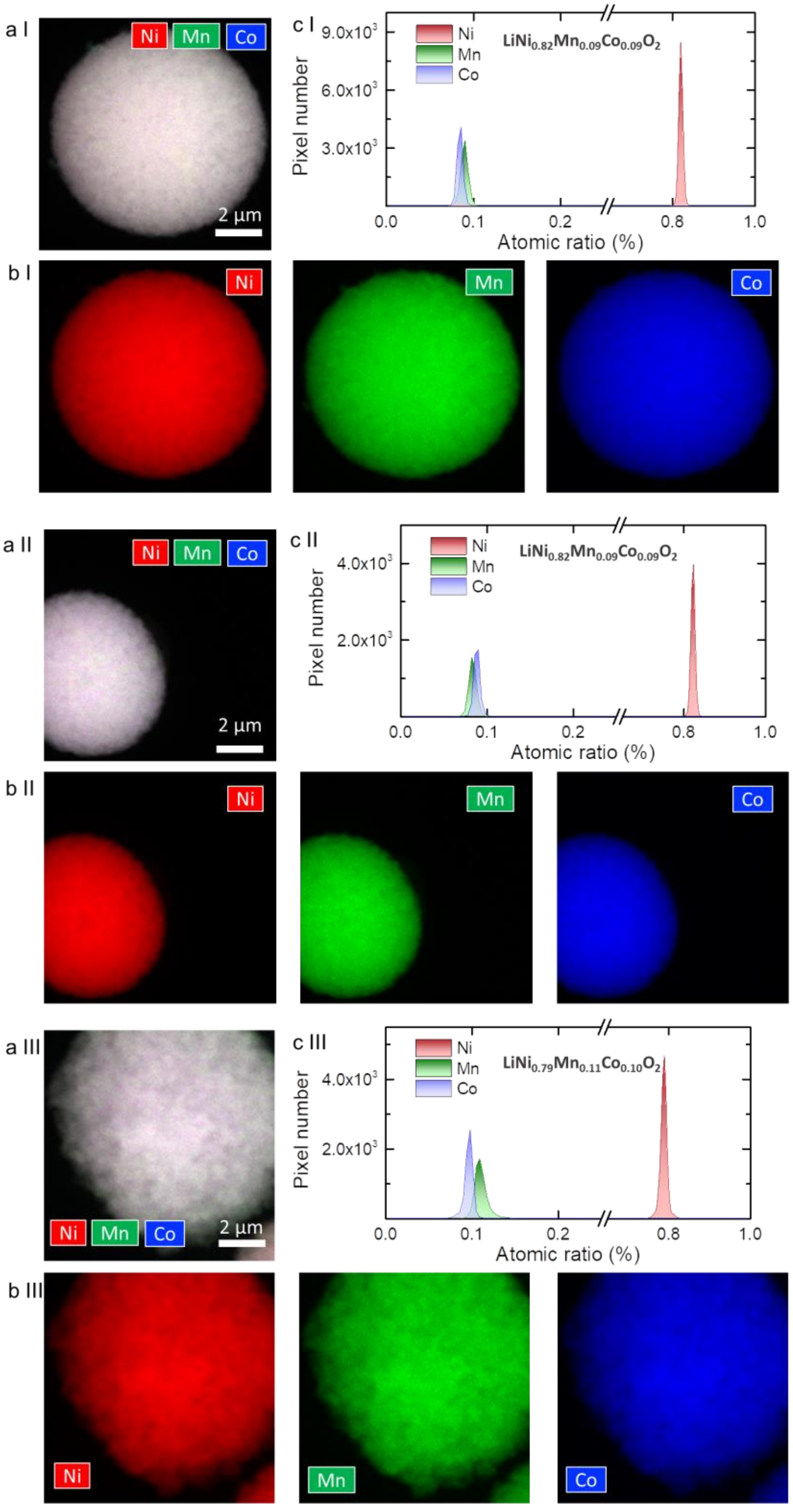

Supplementary Figure 2. Quantitative XRF mapping of Ni, Mn and Co composition in VG-811 secondary particles using the hard X-ray nanoprobes (HXN) at NSLSII. (a I, II, III) color mixed map of the Ni, Mn, Co maps. If the map is white throughout the particle, it means the composition in spatially uniform. (b I, II, III) Ni, Mn and Co concentration maps. (c I, II, III) The histogram distribution of Ni:Mn:Co composition in the three particles. It is worth noting the Ni histogram is

very narrow and sharp indicating there is very little Ni composition variation throughout the particle.

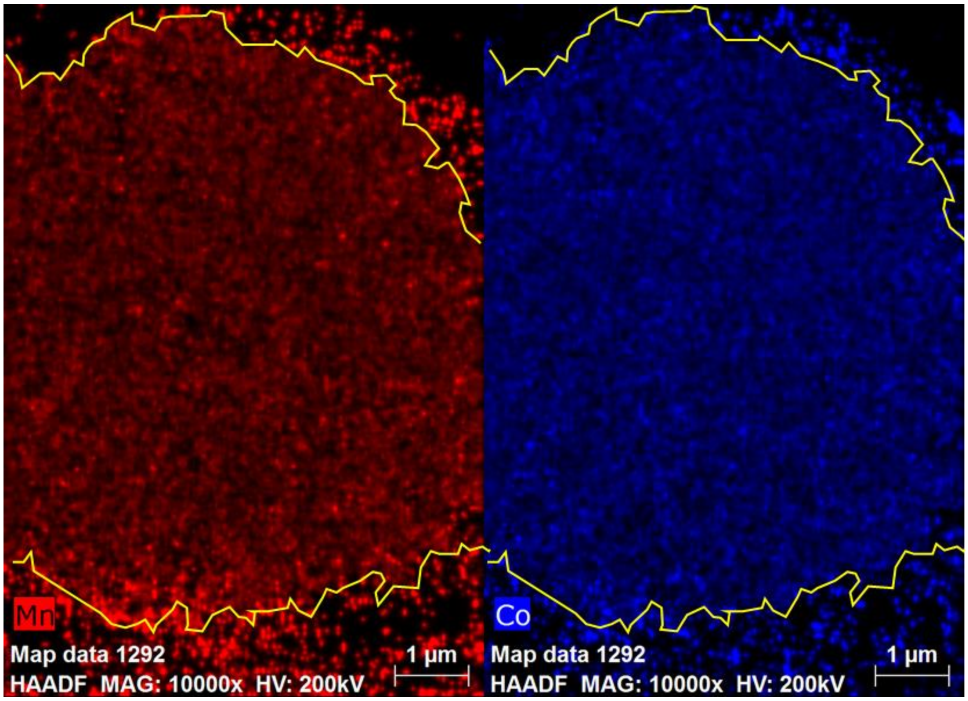

Supplementary Figure 3 STEM-EDS quantitative mapping of Mn and Co

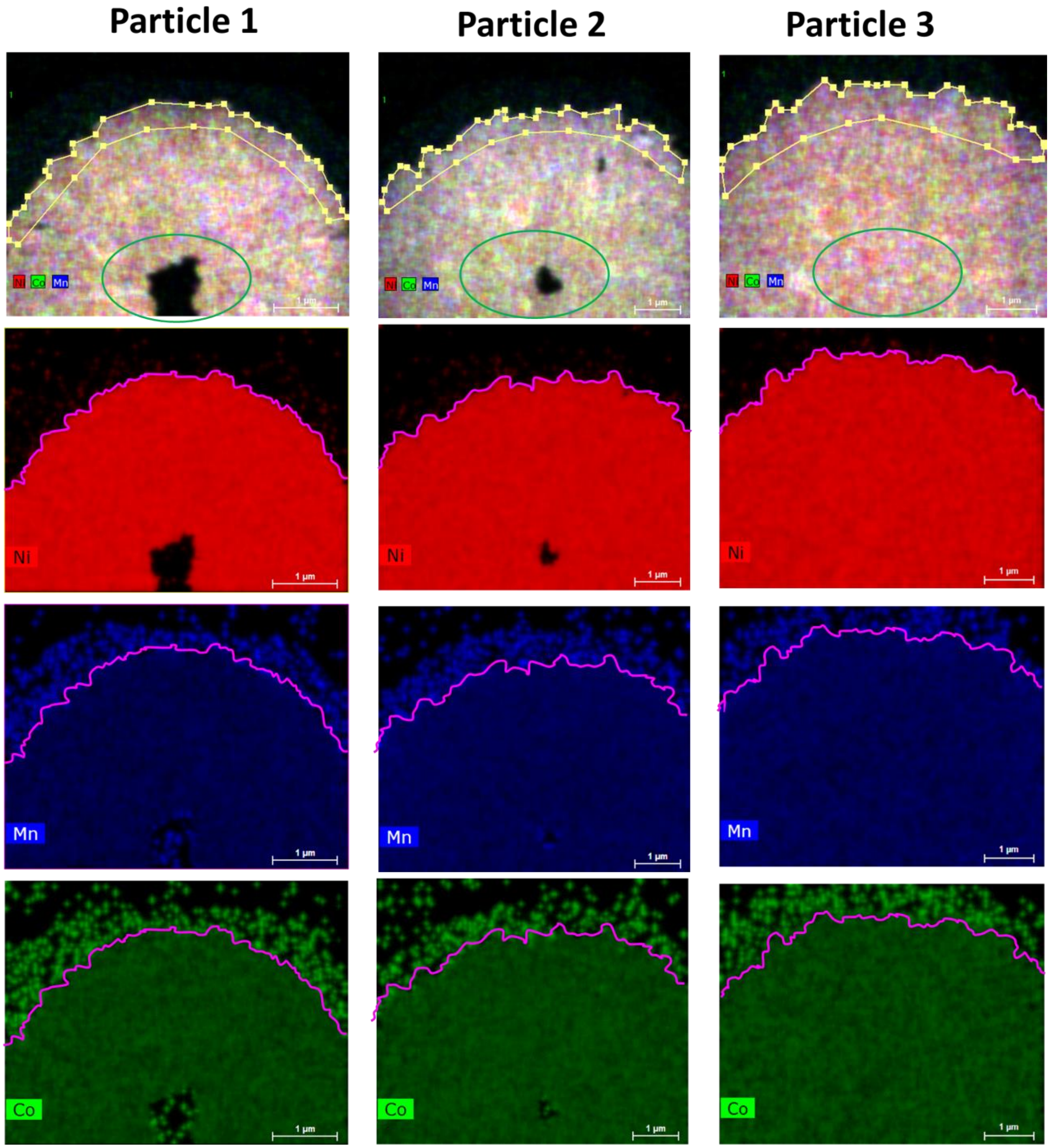

Supplementary Figure 4. Quantitative STEM-EDS mapping of Ni, Mn and Co composition in VG-811 particles. The figures show the quantified Ni, Mn and Co compositions maps. The quantification of the Ni:Mn:Co composition in the surface vs. the interior regions are provided in Supplementary Table 2. (The quantified composition is only valid inside the boundary of the particle as marked by the magenta lines.)

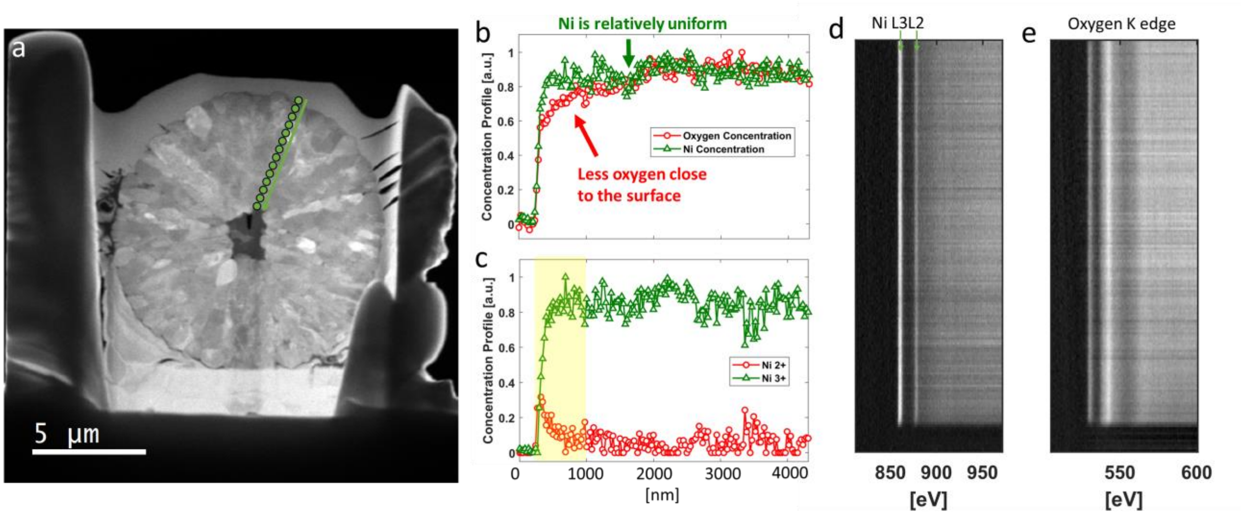

Supplementary Figure 5. Spatially resolved EELS measurement of a pristine VG-811 particle. (a) The overview HAADF-STEM image of the FIB cross section of a VG-811 particle. (b) The Ni and oxygen concentration profile along the profile drawn on (a). (c) The Ni 2+ and Ni 3+ concentration plot. (d and e) The (d) Ni L2,3 and (e) oxygen K edges measured along the profile drawn on (a).

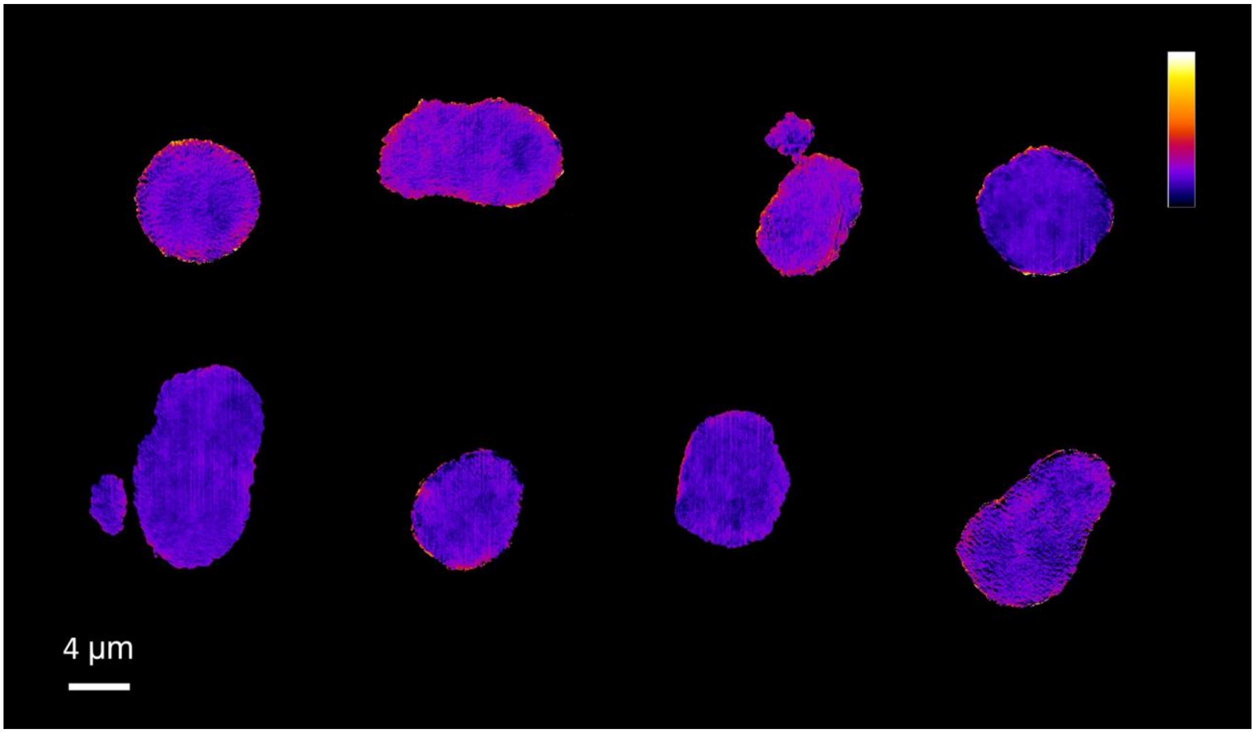

Supplementary Figure 6. Synchrotron hard-X-ray transmission X-ray microscopy 2D XANES mapping of the Ni^{2+} composition in the secondary VG-NMC811 particles.

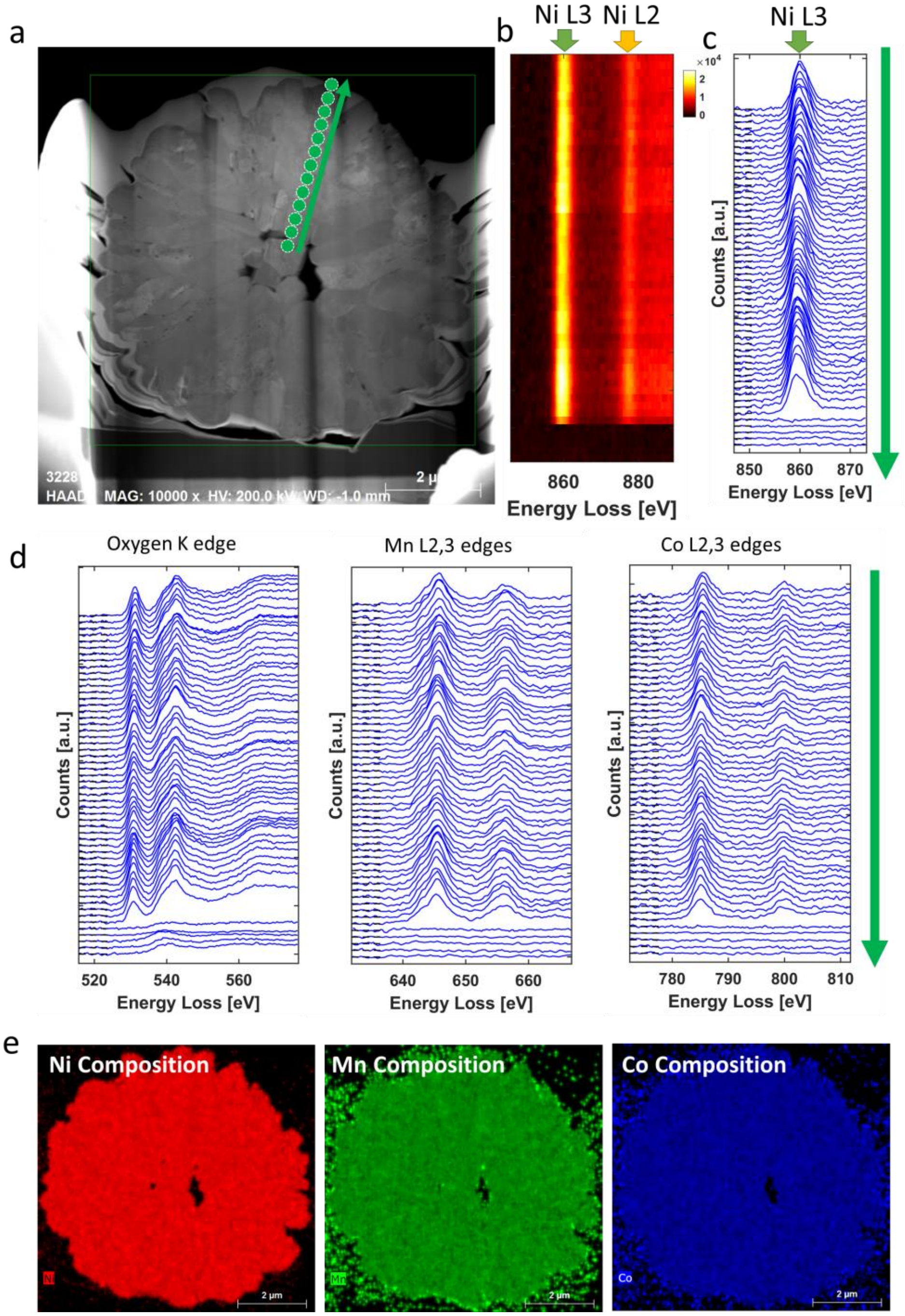

Supplementary Figure 7. Analytical mapping of the conventional NMC811 materials showing both composition and Ni oxidation states are constant through out a secondary particle. (a) The HAADF-STM image of FIB cross-section of the conventional NMC811 material. (b) Spatially resolved Ni L2,3 edges taken along the line profile from the center to the surface of the particle drawn in (a). The data is presented in a 2d intensity plot. (c) Line plots of spatially resolved Ni L3 edge along the line profile. Data in (b) and (c) clearly shows the Ni L edges have no chemical shifts indicating Ni valance state remains constant from the center to the surface in secondary particles. (d) Spatially resolved Oxygen K edge, Mn L2,3 edges, and Co L2,3 edges taken along

the line profile in (a) showing Mn and Co does not have any valance state change. The oxygen pre-peak reflects the O2p-TM3d hybridization. Its intensity typically tracks the population of the TM d-holes. The fact its' position and intensity remains unchanged indicates the transitional metals' bonding oxygen remains constant through out the particle. (e) The STEM-EDS quantification of Ni/Mn/Co composition. It shows Ni composition is constant form the surface to the bulk center and so are Mn and Co.

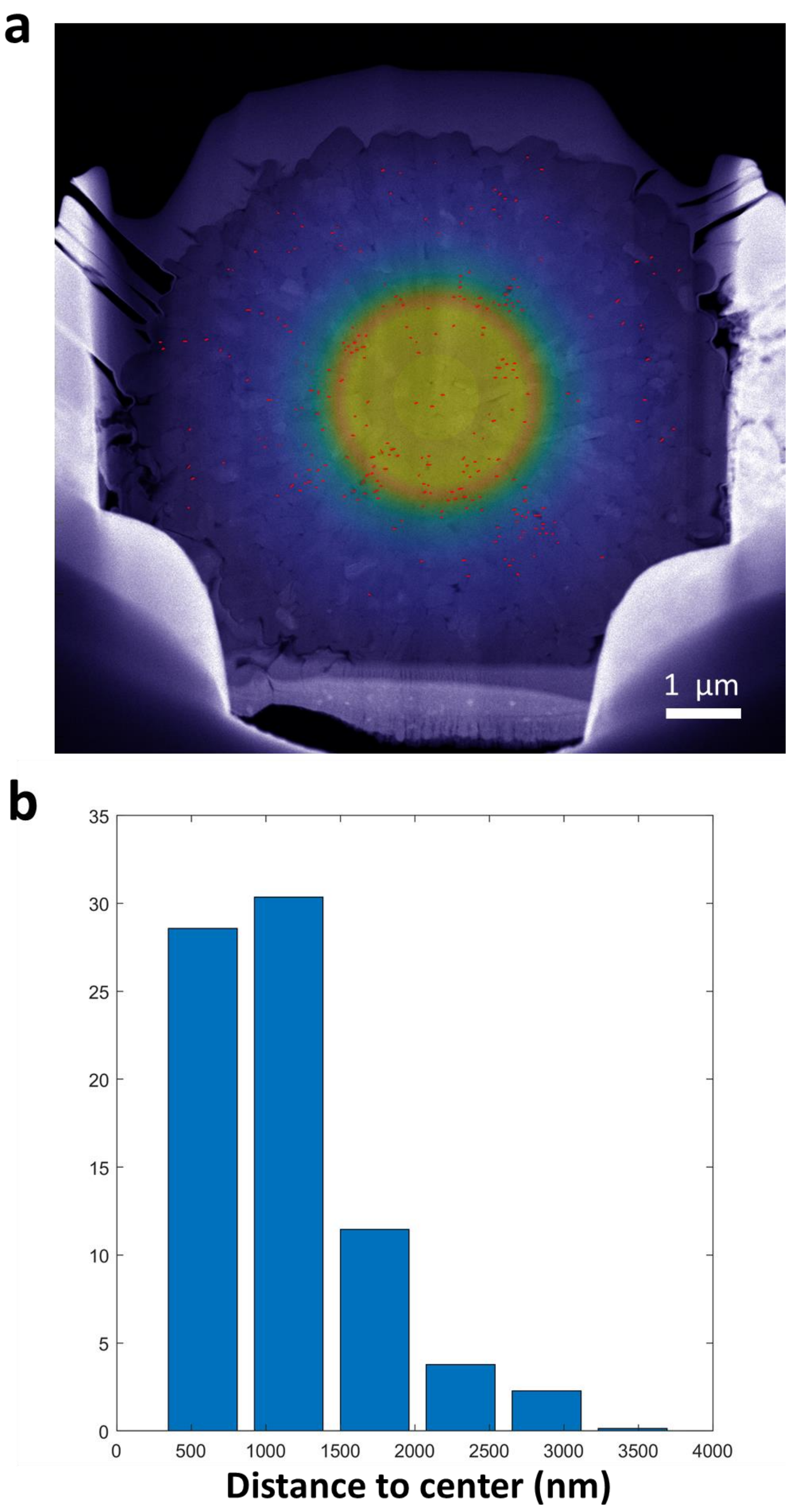

Supplementary Figure 8. HAADF-STEM imaging of a cycled particle prepared by FIB cross section and the analysis of the distribution of nanopores.

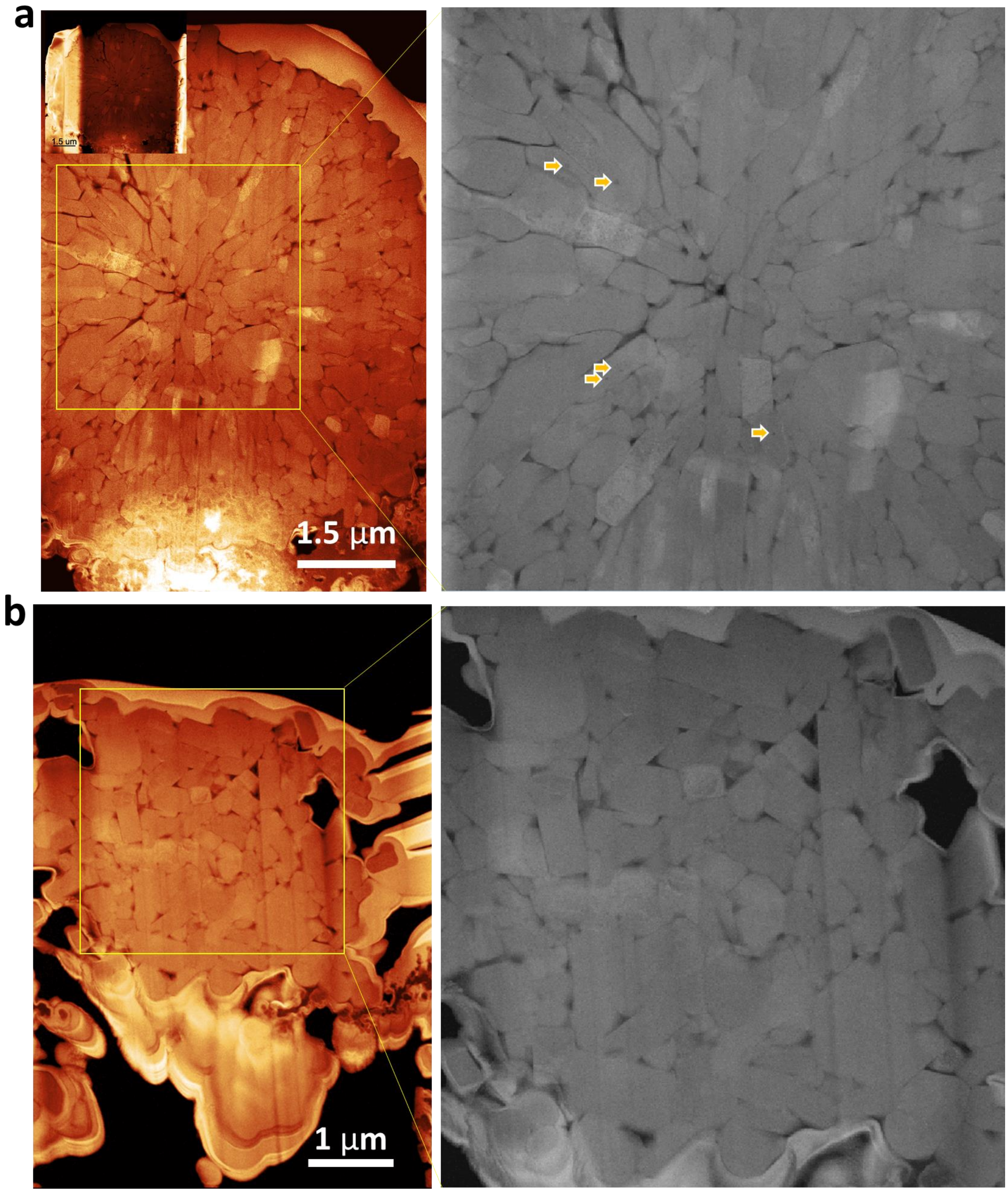

Supplementary Figure 9. HAADF-STEM image of a (**a**) large and a (**b**) small pristine particle prepared by FIB cross section. The pristine particles show few nanopores in the interior of the particles.

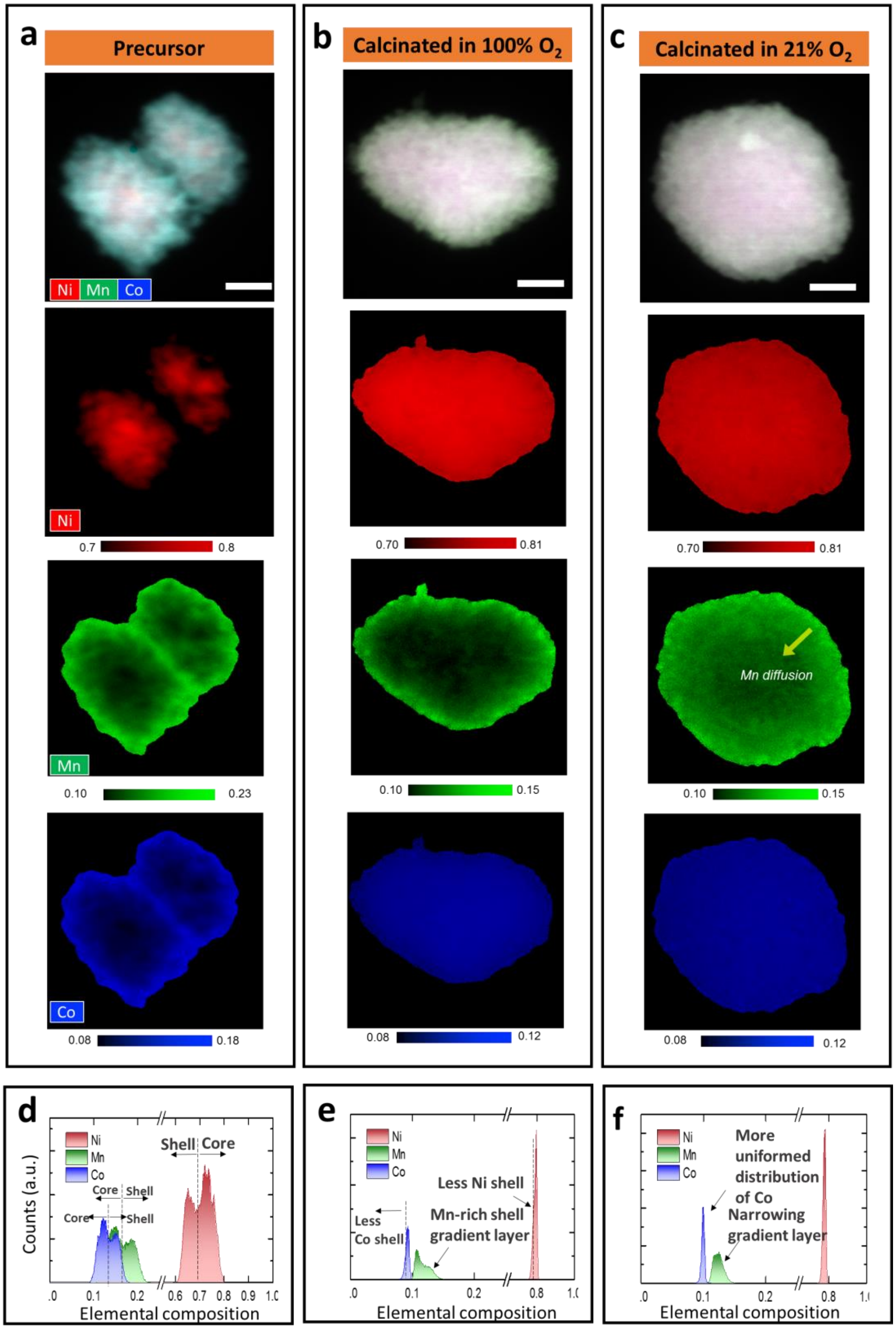

Supplementary Figure 10. X-ray fluorescence composition mapping of (a) Ni, Mn, Co hydroxide precursor (b) NMC811 calcinated in pure O_2 (c) NMC811 calcinated in air; transition

metal composition distribution of (d) Ni, Mn, Co hydroxide precursor (e) NMC811 calcinated in pure O_2. (f) NMC811 calcinated in air (scale bar is 2 μm)

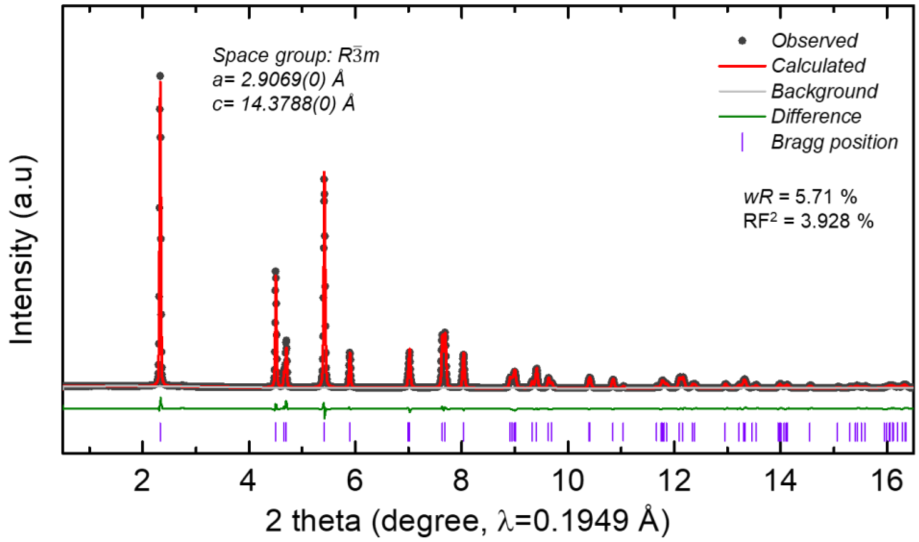

Supplementary Figure 11. XRD of VG-811 precursors calcined in oxygen.

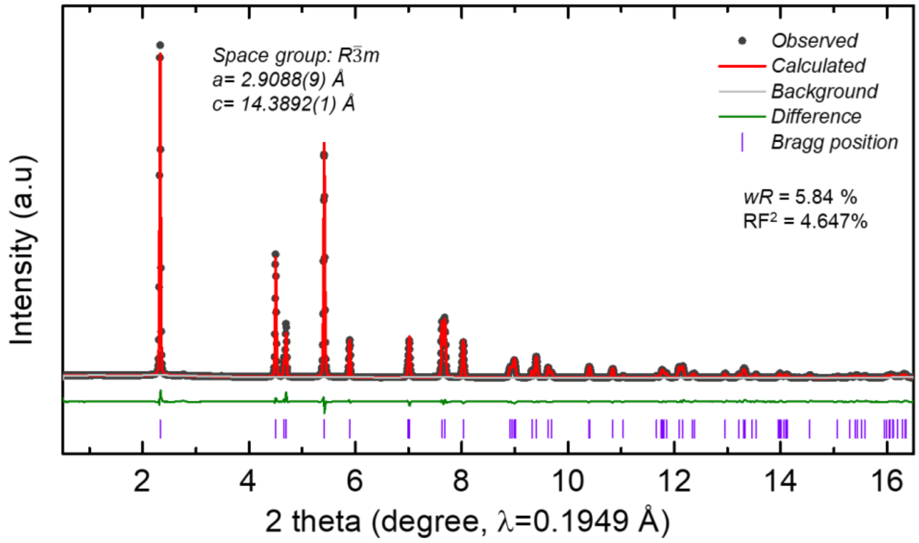

Supplementary Figure 12. XRD of VG-811 precursors calcined in air.

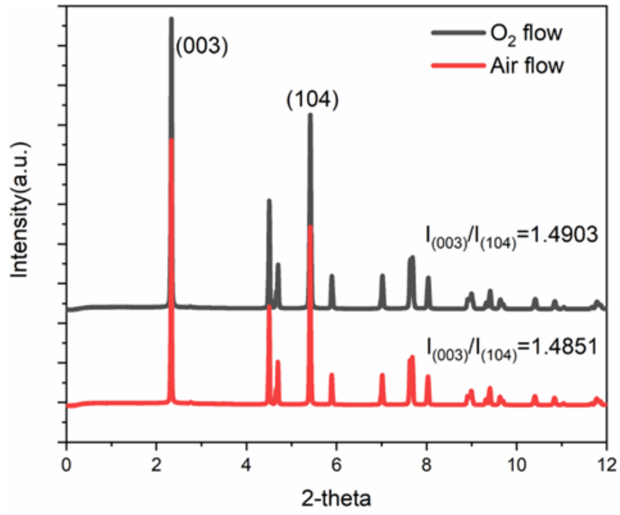

Supplementary Figure 13. X-ray diffraction patterns of VG-NMC811 calcinated in air and pure O_2.
